# Synthesis of gypsogenin derivatives with capabilities to arrest cell cycle and induce apoptosis in human cancer cells

**DOI:** 10.1098/rsos.171510

**Published:** 2018-01-24

**Authors:** Haochao Zhang, Yanling Mu, Fengling Wang, Leling Song, Jie Sun, Yongjun Liu, Jingyong Sun

**Affiliations:** 1 School of Medicine and Life Sciences, University of Jinan-Shandong Academy of Medical Sciences, Jinan 250200, Shandong, China; 2 Institute of Materia Medica, Shandong Academy of Medical Sciences, Jinan 250062, Shandong, China; 3 Key Laboratory for Biotech-Drugs Ministry of Health, Jinan 250062, Shandong, China; 4 Key Laboratory for Rare and Uncommon Diseases of Shandong Province, Jinan 250062, Shandong, China

**Keywords:** gypsogenin derivatives, cytotoxic activity, apoptosis, cell cycle arrest

## Abstract

Thirty-two gypsogenin derivatives were synthesized and screened for their cytotoxic activities. Their structures were established using IR, ^1^H NMR, ^13^C NMR, and LC-MS spectroscopic data. In MTT assays nearly all the compounds displayed good cytotoxicity in the low μM range for several human tumour cell lines (A549, LOVO, SKOV3 and HepG2). Low IC_50_ values were obtained especially for the carboxamides **7a**–**7j**, for an oxime derivative **3** and a (2,4-dinitrophenyl)hydrazono derivative **4**. In particular, the IC_50_ values of compounds **4** (IC_50_ = 2.97 ± 1.13 µΜ) and **7 g** (IC_50_ = 3.59 ± 2.04 µΜ) against LOVO cells were found to be much lower than those of the other derivatives and parent compound. These compounds were submitted to an extensive biological testing and proved compounds **4** and **7 g** to act mainly by an arrest of the tumour cells in the S phase of the cell cycle. In addition, compounds **4** and **7 g** triggered the apoptotic pathway in cancer cells, showing high apoptosis ratios.

## 1. Introduction

Cancer is one of the most challenging problems in medicine. One of the several treatments is chemotherapy, and many chemotherapeutics have been developed so far. Many of them were gained from secondary natural products, for example vinca-alkaloids (from poisonous evergreen, *Catharanthus roseus*), taxeles (diterpenes first derived from the Pacific yew tree, *Taxus brevifolia*) and camptothecins (lactone alkaloids from *Camptotheca acuminata*). Moreover, it was reported that a regular consumption of fruits and herbs helps reduce carcinogenic risk [1,2].

**Figure 1.**
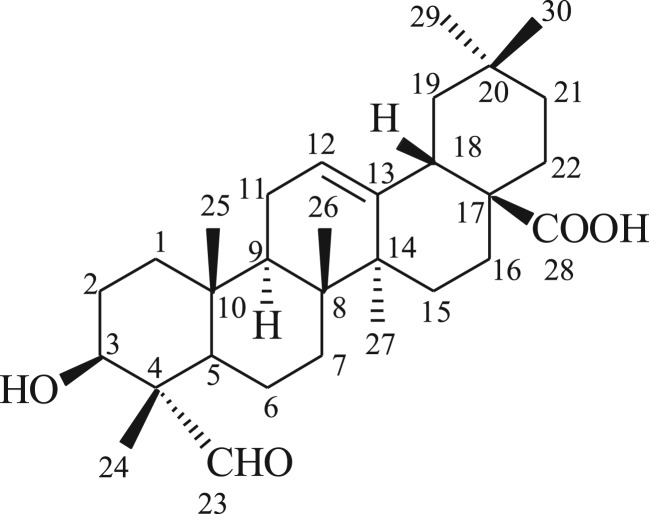
The structure of gypsogenin.

*Gypsophila oldhamiana*, known as ‘xia cao’ in China, belongs to the Caryophyllaceae family [3]. The roots of *Gypsophila* species are an especially rich source of triterpenoid saponins [4,5]. Some triterpenoid saponins from *Gypsophila* have shown a variety of biological activities including anticarcinogenic [6], immunostimulatory [7], cytotoxicity [8–10] and α-glucosidase inhibition activities [11]. Gypsogenin (3-hydroxy-23-oxoolean-12-en-28-oic acid), a natural pentacyclic triterpenoid, has four active sites such as C-3 hydroxyl, ring-C double bond, C-23 aldehyde group and C-28 carboxylic acid, which are amenable for a wide range of chemical transformations. The structure of gypsogenin is shown in figure 1. This valuable compound has been detected and isolated from *Gypsophila oldhamiana.* Many biological activities have been credited to gypsogenin, such as inhibitory activity [12,13], cytotoxicity [14,15], antimicrobial and antiproliferative activities [16]. Of special interest are its cytotoxicity and its antiproliferative properties. In previous study, The C-28 carboxylic acid and C-23 aldehyde group were used to prepare nitrile and different types of esters. These compounds are the first pentacyclic triterpenoids described as a potent AChE-selective inhibitor [17]. The C-23 aldehyde group of gypsogenin was treated with hydroxylamine hydrochloride to provide oxime. This compound triggered the apoptotic pathway in cancer cells, showing high apoptosis ratios [16]. Thus, there is strong evidence that gypsogenin has anti-cancer activity.

Oleanolic acid (3-hydroxy-12-en-28-oic acid), a pentacyclic triterpenoic acid, has three active sites such as C-3 hydroxyl, ring-C double bond and C-28 carboxylic acid. The structure of gypsogenin is very similar to the structure of oleanolic acid. The C-3 hydroxyl and C-28 carboxylic acid of oleanolic acid were used to prepare acetoxy and different types of amide and esters [18,19]. In particular, the different 3-O-acetyl oleanolic acid derived amides have been prepared and screened for their cytotoxic activity [20]. The results reveal that most of the carboxamides displayed good cytotoxicity in the low micromolar range for several human tumour cell lines. These compounds were submitted to an extensive biological testing and proved some compounds to act mainly by an arrest of the tumour cells in the S phase of the cell cycle. In addition, some compounds triggered the apoptotic pathway in cancer cells, showing high apoptosis ratios. These findings make oleanolic acid a promising lead compound for developing new cytotoxic/antitumour active compounds. In previous study, we have never found any references about the cytotoxicity of gypsogenin derived amides. Thus, it is valuable to investigate gypsogenin derived amides.

Gypsogenin aglycone is found at high concentrations in *Gypsophila oldhamiana* [21]; therefore, it can be obtained with ease [22]. In this study, the C-23 aldehyde group has been used to prepare hydrazone and oxime. The different types of esters and amide were designed, and synthesized at C-28 carboxylic acid. In addition, they were evaluated for their cytotoxic activities against four different human cancer cell cultures. More investigations about the mechanism of cell death induced by these gypsogenin derivatives were performed.

## 2. Results and discussion

### 2.1. Chemistry

**Scheme 1.**
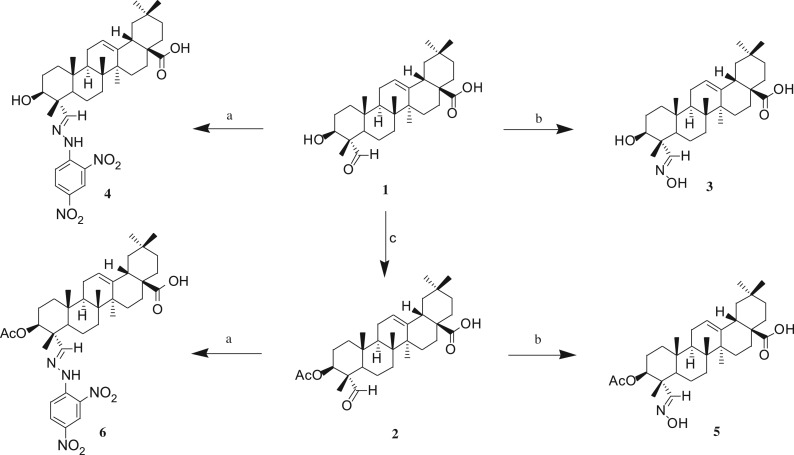
Reagents and conditions: (a) 2,4-dinitrophenylhydrazine, acetic acid, room temperature; (b) hydroxylamine hydrochloride (NH_2_OH·HCl), pyridine, 105°C; (c) acetic anhydride, pyridine, room temperature.

**Scheme 2.**
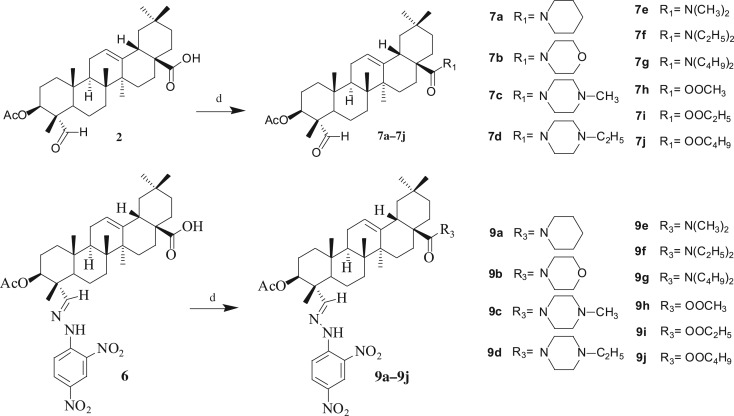
Reagents and conditions: (d) CH_2_Cl_2_, oxalyl chloride, secondary amine or primary alcohol, room temperature.

**Scheme 3.**
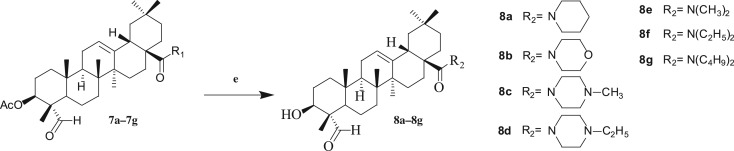
Reagents and conditions: (e) ethyl alcohol absolute, 2.0 mol l^−1^ NaOH, 1.0 mol l^−1^, room temperature.

Thirty-two gypsogenin derivatives were synthesized by a series of reactions as outlined in schemes 1–3. All compounds were obtained in different yields. The gypsogenin (**1**) was obtained by the hydrolysis of the gypsogenin saponin mixtures. For compound **1**, the signals for H-3, H-12 and H-23 could be seen at *δ* 3.95 (1H, dd, *J* = 11.41, 5.15** **Hz), 5.29 (1H, s) and 9.50 (1H, s), respectively. These signals were also evident by their ^13^C NMR spectra showing C-3, C-12 and C-23 at *δ* 71.52, 122.17 and 207.20, respectively. Gypsogenin was acetylated to afford compound **2** in 95.7% yields. In the ^1^H NMR spectrum for compound **2**, the proton signal of H-3 at *δ* 5.23 (dd, *J* = 11.44, 5.16** **Hz) was observed instead of at *δ* 3.95. Compounds **1** and **2** were mixed with hydroxylamine hydrochloride in pyridine at 105°C to provide compounds **3** and **5** in 85.1% and 96.5% yields, respectively. The structures of compounds **3** and **5** were confirmed by their respective ^13^C NMR spectra, which showed the characteristic C-23 carbon signals at *δ* 159.46 and 157.15 respectively. Compounds **1** and **2** were treated with 2,4-dinitrophenylhydrazine in acetic acid at room temperature to obtain compounds **4** and **6** in 88.9% and 86.5% yields, respectively. The ^13^C NMR spectra of compounds **4** and **6** showed the characteristic carbon signals of C-23 at *δ* 163.13 and 158.58 respectively.

Consecutive reactions of the intermediate compounds **2** and **6** with oxalyl chloride and secondary amine in CH_2_Cl_2_ at room temperature gave the corresponding amides **7a–7g** and **9a–9g** in 82–93% and 85–93% yields respectively. Their ^13^C NMR spectra showed the characteristic carbon signals at *δ* 174.45–175.82 attributed to C-28. Under similar conditions, compounds **2** and **6** were coupled with oxalyl chloride and primary alcohols in CH_2_Cl_2_ at room temperature to obtain the corresponding esters **7 h–7j** and **9h–9j** in 89–94% and 89–92% yields, respectively. For compounds **7h–7j** and **9h–9j**, these carbon signals for C-28 could be seen at *δ* 177.42–178.22 respectively.

Compounds **7a–7g** were coupled with NaOH in C_2_H_5_OH at room temperature to afford the corresponding amides **8a–8g** in 82–89% yields. In the ^1^H NMR spectra for compounds **8a–8g**, the proton signal of H-3 at *δ* 5.24–5.26 was converted to *δ* 4.06–4.10. The thirty-two compounds' spectral data are presented in the electronic supplementary material.

### 2.2. Biology

#### 2.2.1. Cytotoxic activity

The 3-(4,5-dimethylthiazol-2-yl)-2,5-diphenyltetrazolium bromide (MTT) assay is a mitochondrial function assay that is based on the ability of viable cells to reduce the MTT to insoluble formazan crystals by mitochondrial dehydrogenase [23]. The anti-cancer activities of the synthesized compounds were studied by an MTT assay using the following human cancer cell lines in cell culture [24]: LOVO (a colon cancer cell line), SKOV3 (an ovary cancer cell line), A549 (a lung cancer cell line) and HepG2 (a liver cancer cell line). Cisplatin along with gypsogenin were taken as reference standards in this study. The IC_50_ values were determined for each compound and cell line. The results of tests are shown in table 1. It is evident that the synthesized compounds showed selectivity in exhibiting significant anti-cancer activity against the four cancer cell lines. Among the tested compounds, **3**, **4** and **7a–7g** were found to be more toxic than others of this series. The structure–activity relationship studies revealed that the introduction of an amide in the 28 position of the target compounds **7a–7g** enhanced the anti-cancer activity. Similarly, the introduction of oxime and 2,4-dinitrophenylhydrazone in the 23 position of the target compounds **3** (IC_50_ = 12.35 ± 1.34 µΜ for LOVO cells) and **4** (IC_50_ = 2.97 ± 1.13 µΜ for LOVO cells) also led to an increase in anti-cancer activity. Our results revealed the superior anti-cancer activity of **4** and **7g** (IC_50_=3.59 ± 2.04 µΜ for LOVO cells) compared to other compounds of the same series.

**Table 1.** Cytotoxic activity of investigated compounds against human cancer cell lines.

	IC_50_ (μM)					IC_50_ (μM)			
compound	A549	LOVO	SKOV3	HepG2	compound	A549	LOVO	SKOV3	HepG2
1	19.60 ± 4.50	15.90 ± 1.87	20.67 ± 3.77	22.18 ± 2.62	8b	28.83 ± 6.78	26.83 ± 2.44	>30	>30
2	30.86 ± 3.26	14.36 ± 2.21	21.20 ± 2.13	24.71 ± 3.15	8c	27.77 ± 5.14	>30	>30	>30
3	17.70 ± 2.49	12.35 ± 1.34	18.51 ± 1.18	19.15 ± 2.21	8d	25.70 ± 2.68	>30	>30	>30
4	3.10 ± 1.14	2.97 ± 1.13	10.04 ± 1.38	9.71 ± 2.06	8e	15.77 ± 2.37	12.36 ± 2.35	25.63 ± 1.14	27.88 ± 2.42
5	28.23 ± 2.04	12.42 ± 1.03	>30	>30	8f	15.03 ± 3.48	11.14 ± 1.87	24.59 ± 2.72	26.91 ± 2.74
6	26.50 ± 1.77	5.31 ± 1.26	>30	>30	8g	14.16 ± 8.70	10.88 ± 1.32	27.38 ± 3.49	28.17 ± 2.88
7a	17.08 ± 2.32	13.49 ± 2.86	16.23 ± 2.35	19.14 ± 2.53	9a	>30	25.98 ± 1.65	>30	>30
7b	15.57 ± 2.17	10.44 ± 2.73	15.74 ± 1.44	17.50 ± 2.48	9b	>30	24.36 ± 1.48	>30	>30
7c	7.32 ± 1.28	7.59 ± 1.63	10.10 ± 2.19	12.33 ± 2.63	9c	>30	24.06 ± 1.17	>30	>30
7d	7.04 ± 1.54	6.18 ± 1.46	9.88 ± 1.54	11.68 ± 1.33	9d	>30	24.65 ± 2.05	>30	>30
7e	11.05 ± 1.87	5.24 ± 1.69	17.34 ± 1.24	16.58 ± 1.84	9e	>30	28.54 ± 2.37	>30	>30
7f	10.55 ± 2.10	4.96 ± 1.64	15.56 ± 1.68	13.14 ± 1.61	9f	>30	25.36 ± 2.43	>30	>30
7g	9.24 ± 1.53	3.59 ± 2.04	13.16 ± 2.96	12.55 ± 1.41	9g	>30	24.55 ± 1.42	>30	>30
7h	8.36 ± 3.97	22.37 ± 3.08	24.77 ± 2.78	>30	9h	>30	>30	>30	>30
7i	8.04 ± 3.65	28.93 ± 2.89	>30	29.61 ± 2.44	9i	>30	>30	>30	>30
7j	9.47 ± 2.81	26.42 ± 3.25	28.74 ± 3.24	>30	9j	>30	>30	>30	>30
8a	>30	>30	>30	>30	cisplatin	0.48 ± 0.21	0.32 ± 0.17	1.55 ± 0.43	0.96 ± 0.51

#### 2.2.2. Morphological observation by acridine orange and ethidium bromide staining

**Figure 2.**
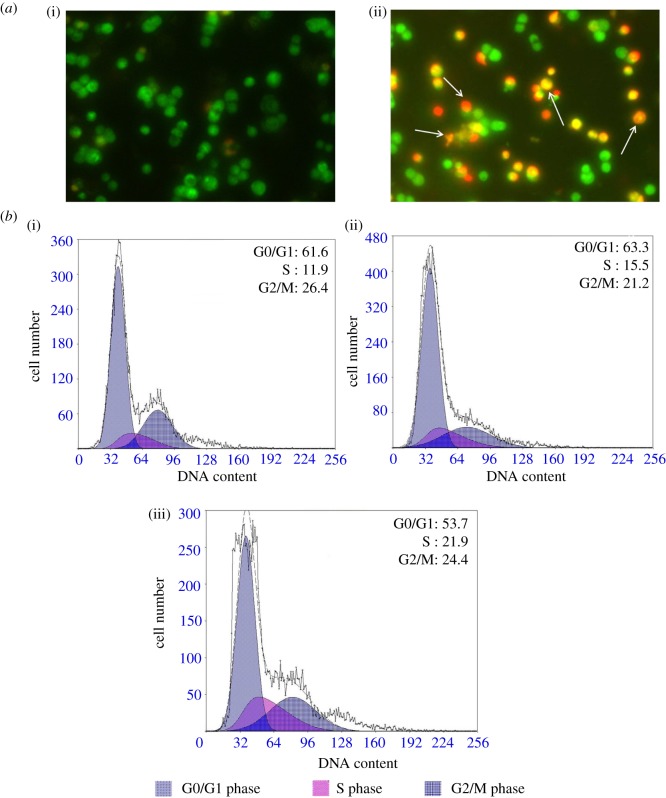
(*a*) Morphological changes in LOVO cells treated with and without compound **4** for 48 h: (i) LOVO control cells; (ii) LOVO cells treated with 5 µM for 48 h followed by morphological observation using AO/EB cell staining method. (*b*) Effect of LOVO on cell cycle progression of colon cancer cells: (i) LOVO control cells; (ii,iii) LOVO cells treated with 5 and 10 µM for 48 h followed by analysis of cell cycle distribution using propidium iodide cell staining method. Cell population in each cell cycle phase was numerically depicted. Data represent one of three independent experiments.

**Figure 3.**
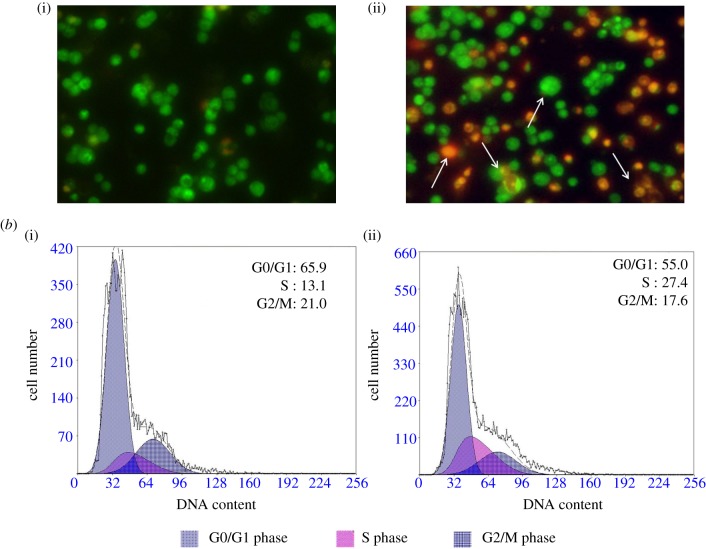
(*a*) Morphological changes in LOVO cells treated with compound **7 g** for 48 h: (i) LOVO control cells; (ii) LOVO cells treated with 5 µM for 48 h followed by morphological observation using AO/EB cell staining method. (*b*) Effect of LOVO on cell cycle progression of colon cancer cells: (i,ii) LOVO cells treated with 5 and 10 µM for 48 h followed by analysis of cell cycle distribution using propidium iodide cell staining method. Cell population in each cell cycle phase was numerically depicted. Data represent one of three independent experiments.

**Figure 4.**
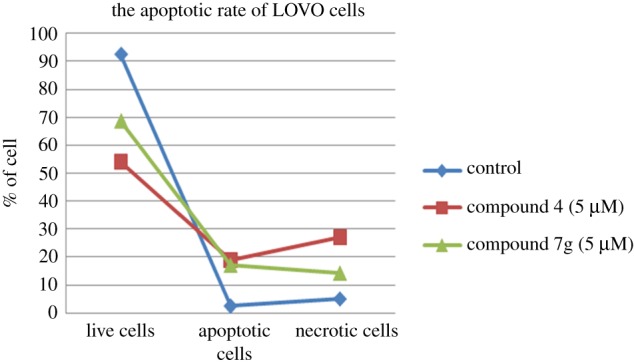
Apoptotic rate of cells treated with and without compounds **4** (5 µM) and **7 g** (5 µM) for 48 h.

The morphological abnormalities induced by compounds **4** and **7g** in LOVO cells were studied under fluorescence microscopy using the acridine orange (AO)/ethidium bromide (EB) staining technique [25]. AO permeates the intact cell membrane and stains the nuclei green, while EB can stain the cells that have lost their membrane integrity and tinge the nucleus red. Intact cells therefore exhibit homogeneous green nuclei, whereas apoptotic cells show condensed or fragmented chromatin. Early apoptotic cells have green nuclei. Late apoptotic or necrotic cells have orange to red nuclei. It can be interpreted from figures 2 and 4 that the control cells showed the normal healthy morphology with intact nuclear architecture and appeared green in colour. Fluorescence microscopic images of LOVO cells treated with compounds **4** and **7g** have clearly demonstrated morphological changes which are the characteristic features of apoptotic cells such as cell shrinkage, membrane blebbing and apoptotic body formation. Compared with spontaneous apoptosis observed in control cells (figure 2), LOVO cells treated for 48** **h with 5** **µM of compound **4** showed a marked increase in percentage of early apoptotic/necrotic cells (figure 2). Cells treated for 48** **h with 5** **µM of compound **7g** showed a significant increase in percentage of late apoptotic/ necrotic cells (figure 4). Apoptotic rates of LOVO cells treated with and without compounds **4** and **7g** for 48** **h are shown in figure 3 and table 2.

**Table 2.** Apoptotic rate of cells treated with and without compounds **4** (5 µM) and **7 g** (5 µM) for 48 h.

	live cells	apoptotic cells	necrotic cells
control	92.5	2.5	5.0
compound **4** (5 µM)	54.1	18.9	27.0
compound **7 g** (5 µM)	68.6	17.1	14.3

#### 2.2.3. Cell cycle analysis by propidium iodide staining

To further investigate the differential growth inhibition mechanism mediated by amide and 2,4-dinitrophenylhydrazone compounds on cell cycle dynamics, the effects of compounds **4** and **7g** on cell cycle progression were determined by propidium iodide (PI) staining method [26]. LOVO cells treated with compounds **4** and **7g** for 48** **h resulted in a marked accumulation of cells in S-phase and a slight reduction in G0/G1 phase as shown. Treatment with the compound **4** at 2.5 µM and 5.0 µM in LOVO cells displayed rise in S-phase population from 11.9% (control) to 15.5% and 21.9% respectively in a dose-dependent manner. For compound **7g**, the ratio of cells in the S phase increased from 11.9% (control) to 13.0% in cells treated with 2.5 µM and 27.4% in cells treated with 5.0 µM. The results of these experiments showed a dose-dependent S-phase arrest in cells treated with compound **4** and compound **7 g**. These results indicated that the compounds **4** and **7g** inhibited the growth of the cancer cells by inhibiting the cell cycle. Especially, the effect of compound **4** is better than compound **7g**. The results are shown in figures 2 and 4.

## 3. Conclusion

In summary, we synthesized thirty-two gypsogenin derivatives in good yield. These compounds were found to be promising lead compounds against the majority of the studied cancer cell lines, exhibiting IC_50_ values much lower than that of the parent compound. The synthesized compounds showed selectivity in exhibiting significant anti-cancer activity against the human cancer cell lines. The structure–activity relationship studies revealed that the introduction of amide in the 28 position and 2,4-dinitrophenylhydrazone in the 23 position of target compounds enhanced the anti-cancer activity. Among all of these compounds, compounds **4** and **7g** could be considered as possible anti-cancer agents as they were shown to affect the cell cycle, causing cell cycle arrest. In addition, compounds **4** and **7g** triggered the apoptotic pathway in cancer cells, showing strong activity in promoting apoptosis.

## 4. Experimental

### 4.1. General

Melting points of all compounds were recorded on an Optimelt-100 automatic melting point apparatus and are uncorrected. IR (KBr) spectra were recorded with a Thermo Nicolet Nexus 670 FT-IR. The NMR spectra were obtained with a Bruker Avance III 600 spectrometer (^1^H: 600 MHz, ^13^C: 150 MHz) with tetramethylsilane as internal standard. Chemical shifts are given in values of ppm and coupling constants in hertz. LC/MS was recorded with an Agilent 1200 capillary spectrometer. Thin-layer chromatography (TLC) was performed on precoated silica gel plates (Qingdao Marine Chemical Industry Factory, Qingdao, China). Column chromatography was carried out using silica gel (200–300 mesh, Qingdao Marine Chemical Industry Factory, Qingdao, China).

### 4.2. General method for the preparation of gypsogenin (1)

The air-dried roots (20 kg) of *Gypsophila oldhamiana* were extracted with H_2_O under refluxing three times at room temperature. The water extract was evaporated *in vacuo* to obtain a yellow mixture (1000 ml). This mixture was hydrolysed with 10% HCl for 72 h before being neutralized with NaOH and extracted with ethyl acetate. The ethyl acetate phase was concentrated to a yellow residue (5.9 kg). Flash chromatography (silica gel, hexane–ethyl acetate, 3 : 1) followed by washing of the crude product (hexane–ethyl acetate, 10 : 1) gave pure gypsogenin (1.3 g) as a white solid.

#### 4.2.1. (3β)-Hydroxy-23-oxoolean-12-en-28-oic acid (gypsogenin (1))

White solid, mp: 273.1–274.2°C, LC/MS (ESI-MS): *m*/*z* = 493.10 (M + 23) (positive ion mode); ^1^H NMR (C_5_D_5_N, 600 MHz) *δ*: 9.50 (1H, s, H-23), 5.29 (1H,br s, 12-H), 3.95 (1H, t, *J* = 7.8 Hz, H-3), 3.19 (1H, dd, *J* = 14.44, 4.63 Hz, H-18), 1.72 (1H, s, H-24), 1.24 (3H, s, H-27), 1.07 (3H, s, H-30), 0.94 (3H, s, H-26), 0.90 (3H, s, H-29), 0.80 (3H, s, H-25); ^13^C NMR (C_5_D_5_N, 150 MHz) *δ*: 207.20 (C-23), 180.10 (C-28), 144.83 (C-13), 122.17 (C-12), 71.52 (C-3), 56.23 (C-4), 47.91 (C-9), 47.61 (C-5), 46.56 (C-17), 46.38 (C-19), 42.14 (C-14), 41.94 (C-18), 40.00 (C-8),38.38 (C-1), 36.15 (C-10), 34.13 (C-21), 33.20 (C-29), 33.47 (C-22), 33.09 (C-7), 30.89 (C-20), 28.19 (C-15), 26.70 (C-2), 26.07 (C-27), 23.79 (C-16), 23.68 (C-30), 23.57 (C-11), 20.97 (C-6), 17.28 (C-26), 15.60 (C-25), 9.60 (C-24).

### 4.3. General procedure for the synthesis of compound 2

Acetic anhydride (15 ml) and dimethylaminopyridine (16.25 mg, 1.33 × 10^−4^ mol) were added to gypsogenin (**1**) (625 mg, 1.33 × 10^−3^ mol) in pyridine (5 ml). The mixture was stirred at room temperature for 24 h. The product was detected with TLC (chloroform–methanol, 100 : 1). After completion of the reaction, the mixture was extracted with ethyl acetate (3 × 20 ml). The organic layer was dried over anhydrous sodium sulfate and evaporated to dryness. The residue was purified by a silica gel column chromatography using chloroform–acetone (100 : 1) to afford the products.

#### 4.3.1. (3β)-Acetyloxy-23-oxoolean-12-en-28-oic acid (2)

White solid, yield: 95.7%, mp: 164.6–166.5°C, IR (KBr) νmax: 3424, 2947, 1735, 1696, 1463, 1372, 1239, 1031, 1009, 728, 684, 634, 601, 514, 415 cm^−1^; LC/MS (ESI-MS): *m/z* = 535.20 (M + 23) (positive ion mode); ^1^H NMR (C_5_D_5_N, 600 MHz) *δ*: 9.52 (1H, s, H-23) 5.48 (1H, br s, 12-H), 5.23 (1H, dd, *J* = 11.44, 5.16 Hz, H-3), 3.30 (1H, dd, *J* = 14.44, 4.63 Hz, H-18), 1.94 (3H, s, CH_3_COO-), 1.28 (1H, s, H-24), 1.18 (3H, s, H-27), 1.02 (3H, s, H-30), 0.97 (3H, s, H-26), 0.96 (3 H, s, H-29), 0.85 (3H, s, H-25); ^13^C NMR (C_5_D_5_N, 150 MHz) *δ*: 204.74 (C-23), 180.18 (C-28), 170.18 (CH_3_COO-),144.89 (C-13), 122.10 (C-12), 73.51 (C-3), 54.65 (C-4), 47.77(C-9), 47.75 (C-5), 46.62 (C-17), 46.43 (C-19), 42.20 (C-14), 41.97 (C-18), 39.98 (C-8), 37.71 (C-1), 35.98 (C-10), 34.20 (C-21), 33.28 (C-29), 33.15 (C-22), 32.36 (C-7), 30.97 (C-20), 28.24 (C-15), 26.27 (C-27), 23.76 (C-30), 23.66 (C-11), 23.61 (C-16), 22.79 (C-2), 20.82 (CH_3_COO-),20.74 (C-6), 17.28 (C-26), 15.44 (C-25),9.75 (C-24).

### 4.4. General procedure for the synthesis of compounds 3 and 5

Gypsogenin (**1**) (50 mg, 1.06 × 10^−4^ mol) and compound **2** (60 mg, 1.17 × 10^−4^ mol) were mixed with hydroxylamine hydrochloride (11.05 mg and 12.19 mg, respectively) and dissolved in pyridine (5 ml). The mixture was stirred at 105°C for 4 h. After stirring, water (10 ml) was added to this mixture, and the mixture was extracted with CHCl_3_ (3 × 15 ml). The combined organic layer was dried over anhydrous sodium sulfate and evaporated to dryness. The crude material was purified by silica gel chromatography using chloroform–methanol (100 : 4) to give compounds **3** and **5**, respectively.

#### 4.4.1. (3β)-Hydroxy-23-(hydroxyimino) olean-12-en-28-oic acid (3)

White solid, yield: 85.1%, mp: 268.0–270.0°C, IR (KBr) νmax: 3939, 3398, 3200, 2941, 1724, 1694, 1458, 1388, 1261, 1180, 956, 816, 734, 591, 476 cm^−1^. LC/MS (ESI-MS): *m/z* = 508.30 (M + 23) (positive ion mode); ^1^H NMR(C_5_D_5_N, 600 MHz) *δ*: 7.76(s, 1H, -CHNOH), 5.51(br s, 1H, H-12), 3.88(dd, *J* = 11.40, 4.2 Hz, 1H, H-3), 3.32(dd, *J* = 13.80, 4.20 Hz, 1H, H-18), 1.47(s, 1H, H-24), 1.29(s, 3H, H-27), 1.02(s, 3H, H-30), 1.01(s, 3H, H-26), 0.97(s, 3H, H-29), 0.95(s, 3H, H-25); ^13^C NMR (C_5_D_5_N, 150 MHz) *δ*: 180.15 (C-28), 159.46 (C-23), 144.88 (C-13), 122.40 (C-12), 75.20 (C-3), 51.96 (C-4), 48.25 (C-9), 47.12 (C-5), 46.66 (C-17), 46.46 (C-19), 42.22 (C-14), 42.04 (C-18), 40.04 (C-8),38.70 (C-1), 36.89 (C-10), 34.22 (C-21), 33.29 (C-20), 33.19 (C-22), 32.87 (C-7), 30.98 (C-29), 28.31 (C-15), 27.15 (C-2), 26.20 (C-27), 23.82 (C-11), 23.78 (C-30), 23.68 (C-16), 20.25 (C-6), 17.42 (C-26), 15.97 (C-25), 12.32 (C-24).

#### 4.4.2. (3β)-Acetyloxy-23-(hydroxyimino) olean-12-en-28-oic acid (5)

White solid, yield: 96.5%, mp: 251.2–252.8°C, IR (KBr) νmax: 3860, 3731, 3260, 2940, 2630, 1731, 1688, 1463, 1370, 1263, 1178, 1035, 1008, 942, 810, 684, 646, 608, 558 cm^−1^; LC/MS (ESI-MS): *m/z* = 528.50 (M + 1) (positive ion mode); ^1^H NMR (C_5_D_5_N, 600 MHz) *δ*: 7.63 (s, 1H, -CHNOH), 5.47 (br s, 1H, H-12), 5.16 (dd, *J* = 11.45, 5.17 Hz, 1H, H-3), 3.30 (dd, *J* = 12.00, 6.00 Hz, 1H, H-18), 1.95 (s, 3H, CH_3_COO-), 1.35 (s, 1H, H-24), 1.27 (s, 3H, H-27), 1.02 (s, 3H, H-30), 0.97 (s, 3H, H-26), 0.97 (s, 3H, H-29), 0.89 (s, 3H, H-25); ^13^C NMR (C_5_D_5_N, 150 MHz) *δ*: 180.14 (C-28), 170.18 (CH_3_COO-), 157.15 (C-23), 144.91 (C-13), 122.21 (C-12), 77.04 (C-3), 52.38 (C-4), 46.63 (C-5), 46.41(C-17), 45.42 (C-19), 42.19 (C-14), 41.98 (C-18), 39.97 (C-8), 37.97 (C-1), 36.76 (C-10), 34.22 (C-21), 33.28 (C-20), 33.19 (C-22), 32.65 (C-7), 30.98 (C-29), 47.99 (C-9), 30.01 (C-2), 28.27 (C-15), 26.21 (C-27), 23.78 (C-30), 23.65 (C-11), 23.29 (C-16), 21.10 (CH_3_COO-), 19.96 (C-6), 17.33 (C-26), 15.80 (C-25), 12.71 (C-24).

### 4.5. General procedure for the synthesis of compounds 4 and 6

Gypsogenin (50 mg, 1.06 × 10^−4^ mol) and compound **2** (60 mg, 1.17 × 10^−4^ mol) were mixed with 2,4-dinitrophenylhydrazine (31.5 mg and 34.8 mg, respectively) and dissolved in acetic acid (5 ml). The mixture was stirred at room temperature for 4 h. After stirring, the water (10 ml) was added to this mixture, and the mixture was extracted with CHCl_3_ (3 × 15 ml). The combined organic layer was dried over anhydrous sodium sulfate and evaporated to dryness. The crude material was purified by silica gel chromatography using chloroform–methanol (100 : 2) to afford compounds **4** and **6**, respectively.

#### 4.5.1. (3β)-Hydroxy-23-[(2,4-dinitrophenyl)hydrazono]olean-12-en-28-oic acid (4)

Yellow solid, yield: 88.9%, mp: 206–207.1°C, IR(KBr) νmax: 3452, 3302, 3101, 2941, 2860, 2853, 1697, 1618, 1589, 1519, 1463, 1425, 1386, 1332, 1277, 1216, 1139, 1075, 958, 920, 832, 712, 646, 580, 526, 454 cm^−1^; LC/MS (ESI-MS): *m/z* = 673.10 (M + 23) (positive ion mode); ^1^H NMR(C_5_D_5_N, 600 MHz) *δ*: 11.54 (s, 1H, -CHNNH-), 9.07 (d, 2.4 Hz, 1H, PhH-3), 8.33 (dd, 9.6, 2.4 Hz, 1H, PhH-5), 8.06 (d, 9.6 Hz, 1H, PhH-6), 7.90 (s, 1H, -CHNNH-), 5.52 (br s, 1H, 12-H), 3.96 (dd, *J* = 11.40, 5.40 Hz, 1H, H-3), 3.32 (dd, *J* = 13.50, 4.50 Hz, 1H, H-18), 1.51 (s, 1H, H-24), 1.27 (s, 3H, H-27), 1.03 (s, 3H, H-30), 1.03 (s, 3H, H-26),0.98 (s, 3H, H-29),0.98 (s, 3H, H-25); ^13^C NMR (C_5_D_5_N, 150 MHz) *δ*: 180.19 (C-28), 163.13 (C-23), 145.55 (Ph-1), 144. 84 (C-13), 137.44 (Ph-4), 129.79 (Ph-5), 129.06 (Ph-2), 123.84 (Ph-6), 122.34 (C-12), 116.95 (Ph-3), 74.99 (C-3), 51.50 (C-4), 49.29 (C-9), 48.32 (C-5), 46.66 (C-17), 46.49 (C-19), 42.20 (C-14), 42.02 (C-18), 40.05 (C-8), 38.64 (C-1), 36.74 (C-10), 36.74 (C-21), 34.22 (C-22), 33.30 (C-29), 33.16 (C-20), 32.75 (C-7), 30.98 (C-15), 28.31 (C-2), 27.15 (C-11), 26.17 (C-27), 23.81 (C-16), 23.77 (C-30), 20.59 (C-6), 17.42 (C-26), 15.94 (C-25), 11.77 (C-24).

#### 4.5.2. (3β)-Acetyloxy-23-[(2,4-dinitrophenyl)hydrazono]olean-12-en-28-oic acid (6)

Yellow solid, yield: 86.5%, mp: 230.2–231.3°C, IR (KBr) νmax: 3295, 3103, 2930, 2861, 2853, 1697, 1618, 1591, 1520, 1460, 1428, 1370, 1333, 1270, 1241, 1071, 1026, 945, 920, 832, 739, 613, 515, 449 cm^−1^; LC/MS (ESI-MS): *m/z* = 692.1 (M − 1) (negative ion mode); ^1^H NMR(CDCl_3_, 600 MHz) *δ*: 11.00 (s, 1H, -CHNNH-), 9.12 (d, 2.4 Hz, 1H, PhH-3), 8.30 (dd, 9.6, 2.4 Hz, 1H, PhH-5), 7.91 (d, 9.6 Hz, 1H, PhH-6), 7.25 (s, 1H, -CHNNH-), 5.29 (br s, 1H, 12-H), 4.92 (dd, *J* = 11.70, 4.20 Hz, 1H, H-3), 2.83 (dd, *J* = 14.10, 4.50 Hz, 1H, H-18), 1.96 (s, 3H, CH_3_COO-), 1.23 (s, 1H, H-24), 1.16 (s, 3H, H-27), 1.04 (s, 3H, H-30), 0.93 (s, 3H, H-26), 0.91 (s, 3H, H-29), 0.77 (s, 3H, H-25); ^13^C NMR (CDCl_3_, 150 MHz) *δ*: 183.73 (C-28), 170.54 (CH_3_COO-), 158.58 (C-23), 145.12 (Ph-1), 143.63 (C-13), 137.84 (Ph-4), 129.97 (Ph-5), 128.99 (Ph-2), 123.53 (Ph-6), 122.17 (C-12), 116.44 (Ph-3), 76.00 (C-3), 52.06 (C-4), 47.80 (C-9), 47.26 (C-5), 46.52 (C-17), 45.78 (C-19), 41.65 (C-14), 40.98 (C-18), 39.60 (C-8), 37.84 (C-1), 36.55 (C-10), 33.75 (C-22), 33.05 (C-20), 32.41 (C-7), 32.22 (C-21), 30.68 (C-29), 29.70 (C-2), 27.63 (C-15), 25.94 (C-30), 23.56 (C-27), 22.95 (C-11), 22.78 (C-16), 21.21 (CH_3_ COO-), 20.11 (C-6), 17.04 (C-26), 15.80 (C-25), 11.98 (C-24).

### 4.6. General procedure for the synthesis of compounds 7a–7j

Oxalyl chloride (2.0 × 10^−1^ ml, 2.36 × 10^−3^ mol) was added to compound **2** (40 mg, 7.8 × 10^−5^ mol) in CH_2_Cl_2_ (3 ml). The mixture was allowed to stir for 12 h at room temperature. After completion, the reaction mixture was neutralized with Et_3_N and evaporated to dryness. To a stirred solution of the mixture in dry CH_2_Cl_2_ (3 ml) was added piperidine (4.0 × 10^−2^ ml, 4.04 × 10^−4^ mol). The stirring was continued for 8 h at room temperature. After CH_2_Cl_2_ evaporation, water (5 ml) was added to this mixture, and the mixture was extracted with ethyl acetate (3 × 5 ml). The combined organic layer was dried over anhydrous sodium sulfate and evaporated to dryness. The crude material was purified by silica gel chromatography using chloroform–methanol (100 : 2) to afford compound **7a**. Compounds **7b–7j** were prepared as **7a**.

#### 4.6.1. *N*-Piperidy-(3β)-acetyloxy-23-oxoolean-12-en-28-amide (7a)

White solid, yield: 85%, mp: 117.2–118.1°C, IR (KBr) νmax: 3435, 3358, 2937, 2854, 1735, 1630, 1465, 1446, 1371, 1240, 1028, 1002, 800, 739, 679, 641, 602, 526, 471 cm^−1^; LC/MS (ESI-MS): *m/z* = 580.50 (M + 1) (positive ion mode); ^1^H NMR (C_5_D_5_N, 600 MHz) *δ*: 9.53 (s, 1H, H-23), 5.44 (br s, 1H, 12-H), 5.24 (dd, *J* = 11.10, 5.70 Hz, 1H, H-3), 3.59 (m, 4H, -N(CH_2_)_2_-(CH_2_)_3_), 3.42 (dd, *J* = 14.70, 3.90 Hz, 1H, H-18), 1.94 (s, 3H, CH_3_COO-), 1.50 (m, 4H, -N(CH_2_)_2_-(CH_2_)_2_-CH_2_), 1.31 (m, 2H, -N(CH_2_)_4_-CH_2_), 1.27 (s, 1H, H-24), 1.22 (s, 3H, H-27), 0.98 (s, 3H, H-30), 0.97 (s, 3H, H-26), 0.92 (s, 3H, H-29),0.91 (s, 3H, H-25); ^13^C NMR (C_5_D_5_N, 150 MHz) *δ*: 204.79 (C-23), 174.45 (C-28), 170.19 (CH_3_COO-), 145.61 (C-13), 121.29 (C-12), 73.54 (C-3), 54.69 (C-4), 47.89 (C-9), 47.86 (C-5), 47.54 (-N(CH_2_)_2_-(CH_2_)_3_), 46.82 (C-17), 46.65 (C-19), 44.18 (C-14), 42.28 (C-18), 39.84 (C-8), 37.75 (C-1), 36.05 (C-10), 36.05 (C-21), 34.30 (C-22), 33.20 (C-20), 32.59 (C-7), 30.58 (C-29), 29.98 (C-2), 28.32 (C-15), 26.51 (-N(CH_2_)_2_-(CH_2_)_2_-CH_2_), 26.13 (C-11), 25.05 (-N(CH _2_)_4_-CH_2_), 24.19 (C-16), 23.64 (C-30), 22.81 (C-27), 20.83 (CH_3_COO-), 20.83 (C-6), 17.20 (C-26), 15.57 (C-25), 9.78 (C-24).

#### 4.6.2. *N*-Morpholino-(3β)-acetyloxy-23-oxoolean-12-en-28-amide (7b)

White solid, yield: 86.7%, mp: 129.8–135.1°C, IR(KBr) νmax: 3430, 2948, 2853, 2695, 1734, 1635, 1457, 1298, 1238, 1183, 1118, 1025, 975, 893, 849, 745, 690, 657, 526, 460 cm^−1^; LC/MS (ESI-MS): *m/z* = 582.50 (M + 1) (positive ion mode); ^1^H NMR (C_5_D_5_N, 600 MHz) *δ*: 9.54 (s, 1H, H-23), 5.44 (br s, 1H, 12-H), 5.25 (dd, *J* = 11.10, 5.70 Hz, 1H, H-3), 3.73–3.80 (m, 4H, -N(CH_2_)_2_-(CH_2_)_2_O), 3.69–3.72 (m, 4H, -N(CH_2_)_2_-(CH_2_)_2_O), 3.39 (dd, *J* = 15.00, 5.40 Hz, 1H, H-18), 1.95 (s, 3H, CH_3_COO-), 1.26 (s, 1H, H-24), 1.22 (s, 3H, H-27), 0.98 (s, 3H, H-30), 0.97 (s, 3H, H-26), 0.91 (s, 3H, H-29), 0.89 (s, 3H, H-25); ^13^C NMR (C_5_D_5_N, 150 MHz) *δ*: 204.84 (C-23), 175.03 (C-28), 170.23 (CH_3_COO-), 145.34 (C-13), 121.51 (C-12), 73.56 (C-3), 67.17 (-N(CH_2_)_2_-(CH_2_)_2_O), 54.69 (C-4), 47.86 (C-9), 47.57 (C-5), 47.57 (-N(CH_2_)_2_-(CH_2_)_2_O), 46.63 (C-17), 46.39 (C-19), 44.08 (C-14), 42.28 (C-18), 39.84 (C-8), 37.75 (C-1), 36.04 (C-10), 36.04 (C-21), 34.17 (C-22), 33.17 (C-20), 32.51 (C-7), 30.56 (C-29),30.04 (C-2), 28.16 (C-15), 26.16 (C-11), 24.14 (C-16), 23.63 (C-30), 22.80 (C-27), 20.84 (CH_3_COO-) ,20.82 (C-6), 17.11 (C-26), 15.57 (C-25), 9.78 (C-24).

#### 4.6.3. *N*-(1-Methyl-piperazinyl)-(3β)-acetyloxy-23-oxoolean-12-en-28-amide (7c)

White solid, yield: 92.4%, mp: 129.8–131.3°C, IR(KBr) νmax: 3413, 2945, 2854, 2794, 2690, 1732, 1630, 1460, 1405, 1293, 1252, 1141, 1031, 1001, 893, 821, 772, 745, 690, 657, 531, 471 cm^−1^; LC/MS (ESI-MS): *m/z* = 595.5 (M + 1) (positive ion mode); ^1^H NMR (C_5_D_5_N, 600 MHz) *δ*: 9.54 (s, 1H, H-23), 5.44 (br s, 1H, 12-H), 5.25 (dd, *J* = 12.00, 6.00 Hz, 1H, H-3), 3.76–3.83 (m, 4H,-N(CH_2_)_2_(CH_2_)_2_NCH_3_), 3.39 (dd, *J* = 12.00, 4.20 Hz, 1H, H-18), 2.38–2.44 (m, 4H, -N(CH_2_)_2_ (CH_2_)_2_ NCH_3_), 2.24 (s, 3H,-N(CH_2_)_4_NCH_3_), 1.95 (s, 3H, CH_3_COO-), 1.26 (s, 1H, H-24), 1.22 (s, 3H, H-27), 0.98 (s, 3H, H-30), 0.97 (s, 3H, H-26), 0.90 (s, 3H, H-29), 0.90 (s, 3H, H-25); ^13^C NMR (C_5_D_5_N, 150 MHz) *δ*: 204.78 (C-23), 174.73 (C-28), 170.18 (CH_3_COO-), 145.44 (C-13), 121.38 (C-12), 73.49 (C-3), 55.45 (-N(CH_2_)_2_(CH_2_)_2_NCH_3_), 54.70 (C-4), 47.84 (C-9), 47.78 (C-5), 47.51 (C-17), 46.67 (C-19), 45.89 (-N(CH_2_)_4_NCH_3_), 45. 54 (-N (CH_2_)_2_(CH_2_)_2_NCH_3_), 42.22 (C-14), 42.22 (C-18), 39.81 (C-8), 37.69 (C-1), 36.01 (C-10), 36.01 (C-21), 34.22 (C-22), 33.20 (C-20), 32.50 (C-7), 30.57 (C-29), 30.03 (C-2), 28.21 (C-15), 26.14 (C-11), 24.13 (C-16), 23.62 (C-30), 22.78 (C-27), 20.85 (CH_3_COO-), 20.80 (C-6), 17.15 (C-26), 15.55 (C-25), 9.77 (C-24).

#### 4.6.4. *N*-(1-Ethyl-piperazinyl)-(3β)-acetyloxy-23-oxoolean-12-en-28-amide (7d)

White solid, yield: 91.7%, mp: 126.2–128.1°C, IR (KBr) νmax: 3534, 3424, 3194, 2943, 2849, 2805, 2695, 1732, 1630, 1455, 1407, 1378, 1251, 1141, 1163, 1023, 938, 904, 810, 767, 745, 684, 635, 602, 526, 460 cm^−1^; LC/MS (ESI-MS): *m/z* = 609.5 (M + 1) (positive ion mode); ^1^H NMR (C_5_D_5_N, 600 MHz) *δ*: 9.53 (s, 1H, H-23), 5.44 (brs, 1H, 12-H), 5.25 (dd, *J* = 11.10, 5.70 Hz, 1H, H-3), 3.44 (dd, *J*= 15.60, 4.20 Hz, 1H, H-18), 3.74–3.81 (m, 4H, -N(CH_2_)_2_(CH_2_)_2_ NC_2_ H_5_), 2.37–2.44 (m, 4H, -N(CH_2_)_2_(CH_2_)_2_NC_2_H_5_), 2.32 (q, 2H, -N (CH_2_)_4_ NCH_2_CH_3_), 1.94 (s, 3H, CH_3_COO-), 1.27 (s, 1H, H-24), 1.22 (s, 3H, H-27), 1.04 (t, 3H, -N(CH_2_)_4_NCH_2_CH_3_), 0.99 (s, 3H, H-30), 0.97 (s, 3H, H-26), 0.92 (s, 3H, H-29), 0.91 (s, 3H, H-25); ^13^C NMR (C_5_D_5_N, 150 MHz) *δ*: 204.75 (C-23), 174.68 (C-28), 170.15 (CH_3_C OO-), 145.50 (C-13), 121. 41 (C-12), 73.54 (C-3), 54.69 (-N(CH _2_)_2_(CH_2_)_2_NC_2_H_5_), 53.41 (-N(CH_2_)_2_(CH_2_)_2_NC_2_H_5_), 52.38 (C-4), 47.90 (C-9), 47.86 (C-5), 47.55 (C-17), 46.72 (C-19), 45.89 (-N(CH_2_)_4_ NCH_2_CH_3_), 42.12 (C-14), 42.28 (C-18), 39.87 (C-8), 37.75 (C-1), 36.06 (C-10), 36.06 (C-21), 34.26 (C-22), 33.21 (C-20), 32.57 (C-7), 30.59 (C-29), 30.13 (C-2), 28.27 (C-15), 26.18 (C-11), 24.17 (C-16), 23.65 (C-30), 22.82 (C-27), 20.83 (CH_3_COO-), 20.83 (C-6), 17.22 (C-26), 15.57 (C-25), 12.30 (-N(C H_2_)_4_ NCH_2_CH_3_), 9.78 (C-24).

#### 4.6.5. *N,N*-Dimethyl-(3β)-acetyloxy-23-oxoolean-12-en-28-amide (7e)

White solid, yield: 82.1%, mp: 184.1–186.4°C, IR (KBr) νmax: 3446, 2947, 2865, 2695, 1735, 1629, 1465, 1370, 1239, 1141, 1029, 975, 904, 816, 684, 641, 564, 526 cm^−1^; LC/MS (ESI-MS): *m/z* = 540.40 (M + 1) (positive ion mode); ^1^H NMR (C_5_D_5_N, 600 MHz) *δ*: 9.54 (s, 1H, H-23), 5.42 (br s, 1H, 12-H), 5.25 (dd, *J* = 11.1,5.7 Hz, 1H, H-3), 3.43 (dd, *J* = 13.5, 3.9 Hz, 1H, H-18), 3.01 (s, 6H,-N(CH_3_)_2_), 1.95 (s, 3H, CH_3_COO-), 1.25 (s, 1H, H-24), 1.21 (s, 3H, H-27), 0.97 (s, 3H, H-30), 0.96 (s, 3H, H-26), 0.88 (s, 3H, H-29), 0.84 (s, 3H, H-25); ^13^C NMR (C_5_D_5_N, 150 MHz) *δ*: 204.74 (C-23), 175.82 (C-28), 170.16 (CH_3_COO-), 145.51 (C-13), 121.47 (C-12), 73.54 (C-3), 54.70 (C-4), 47.88 (C-9), 47.86 (C-5), 47.57 (C-17), 46.58 (C-19), 43.84 (C-14), 42.31 (C-18), 39.76 (C-8), 38.61 (-N(CH_3_)_2_), 37.72 (C-1), 36.04 (C-10), 36.04 (C-21), 34.13 (C-22), 33.25 (C-20), 32.42 (C-7), 30.65 (C-29), 29.89 (C-2), 27.99 (C-15), 26.32 (C-11), 24.19 (C-16), 23.63 (C-30), 22.81 (C-27), 20.83 (CH_3_COO-), 20.79 (C-6), 17.03 (C-26), 15.51 (C-25), 9.78 (C-24).

#### 4.6.6. *N*,*N*-Diethyl-(3β)-acetyloxy-23-oxoolean-12-en-28-amide (7f)

White solid, yield: 85.5%, mp: 175.8–176.1°C, IR (KBr) νmax: 2939, 2869, 2695, 1732, 1624, 1466, 1406, 1378, 1253, 1139, 1031, 936, 893, 739, 635, 608, 558, 526, 471 cm^−1^; LC/MS (ESI-MS): *m/z* = 568.6 (M + 1) (positive ion mode); ^1^H NMR (C_5_D_5_N, 600 MHz) *δ*: 9.55 (s, 1H, H-23), 5.46 (br s, 1H, 12-H), 5.26 (dd, *J* = 10.8, 6.0 Hz, 1H, H-3), 3.34–3.44 (m, 4H, -N(CH_2_-CH_3_)_2_), 3.43 (dd, *J* = 14.45, 4.62 Hz, 1H, H-18), 1.95 (s, 3H, CH_3_COO-), 1.27 (s, 1H, H-24), 1.22 (s, 3H, H-27), 1.15 (t, 6H, -N(CH_2_CH_3_)_2_), 0.97 (s, 3H, H-30), 0.96 (s, 3H, H-26), 0.92 (s, 3H, H-29), 0.89 (s, 3H, H-25); ^13^C NMR (C_5_D_5_ N,150 MHz) *δ*: 204.81 (C-23), 174.75 (C-28), 170.20 (CH_3_COO-), 145.61 (C-13), 121.22 (C-12), 73.50 (C-3), 54.72 (C-4), 47.83 (C-9), 47.81 (C-5), 47.65 (C-17), 47.06 (C-19), 44.13 (-N(CH_2_CH_3_)_2_), 42.25 (C-14), 42.36 (C-18), 39.92 (C-8), 37.74 (C-1), 36.03 (C-10), 36.03 (C-21), 34.41 (C-22), 33.18 (C-20), 32.64 (C-7), 30.56 (C-29), 30.03 (C-2), 28.41 (C-15), 25.95 (C-11), 24.11 (C-16), 23.66 (C-30), 22.79 (C-27), 20.85 (CH_3_COO-), 20.83 (C-6), 17.46 (C-26), 15.57 (C-25), 13.61 (-N(CH_2_CH_3_)_2_), 9.79 (C-24).

#### 4.6.7. *N*,*N*-(*n*-Dibutyl)-(3β)-acetyloxy-23-oxoolean-12-en-28-amide (7g)

White solid, yield: 82.4%, mp: 169.7–171.1°C, IR (KBr) νmax: 3424, 2947, 1735, 1696, 1463, 1372, 1239, 1031, 1009, 728, 684, 634, 601, 514, 415 cm^−1^; LC/MS (ESI-MS): *m/z* = 624.6 (M + 1) (positive ion mode); ^1^H NMR (C_5_D_5_N, 600 MHz) *δ*: 9.52 (s, 1H, H-23), 5.46 (br s, 1H, 12-H), 5.24 (dd, *J* = 11.4, 5.7 Hz, 1H, H-3), 3.43 (dd, *J* = 14.44, 4.63 Hz, 1H, H-18), 3.35 (br s, 4H, -N(CH_2_-C_3_H_7_)_2_), 1.94 (s, 3H, CH_3_COO-), 1.31–1.36 (m, 8H, -N(CH_2_C_2_H_4_CH_3_)_2_), 1.29 (s, 1H, H-24), 1.20 (s, 3H, H-27), 0.98 (s, 3H, H-30), 0.97 (s, 3H, H-26), 0.96 (s, 3H, H-29), 0.95 (t, 6H, -N(C_3_H_6_CH_3_)_2_), 0.90 (s, 3H, H-25); ^13^C NMR (C_5_D_5_N, 150 MHz) *δ*: 204.73 (C-23), 174.98 (C-28), 170.14 (CH_3_COO-), 145.68 (C-13), 121.29 (C-12), 73.54 (C-3), 54.69 (C-4), 47.90 (C-9), 48.42, 44.19 (-N(CH_2_C_3_H_7_)_2_), 47.87 (C-5), 47.87 (C-17), 47.18 (C-19), 44.19 (C-14), 42.44 (C-18), 40.00 (C-8), 37.78 (C-1), 36.07 (C-10), 36.07 (C-21), 34.42 (C-22), 33.16 (C-20), 32.69 (C-7), 30.58 (C-29), 30.01 (C-2), 29.94, 26.03 (-N(CH_2_CH_2_C_2_H_5_)_2_), 28.55 (C-15), 26.03 (C-11), 24.23 (C-16), 23.69 (C-30), 22.82 (C-27), 20.82 (CH_3_COO-), 20.71 (C-6), 19.59, 14.20 (-N(C_2_ H_4_CH_2_CH_3_)_2_), 17.52 (C-26), 15.58 (C-25), 14.20 (-N(C_3_H_6_CH_3_)_2_), 9.79 (C-24).

#### 4.6.8. Methyl (3β)-acetyloxy-23-oxoolean-12-en-28-oate (7h)

White solid, yield: 93.5%, mp: 160.5–162.2°C, IR (KBr) νmax: 3424, 2950, 2879, 2950, 1720, 1446, 1378, 1244, 1165, 1032, 969, 882, 810, 745, 635, 564, 465 cm^−1^; LC/MS (ESI-MS): *m/z* = 549.3 (M + 23) (positive ion mode); ^1^H NMR (C_5_D_5_N, 600 MHz) *δ*: 9.52 (s, 1H, H-23), 5.39 (br s, 1H, 12-H), 5.24 (dd, *J* = 11.40, 5.40 Hz, 1H, H-3), 3.72 (s, 3H, -OCH_3_); 3.11 (dd, *J* = 14.10, 4.50 Hz, 1H, H-18), 1.94 (s, 3H, CH_3_COO-),1.22 (s, 1H, H-24), 1.21 (s, 3H, H-27), 0.95 (3H, s, H-30), 0.94 (s, 3H, H-26), 0.89 (s, 3H, H-29), 0.80 (s, 3H, H-25); ^13^C NMR (C_5_D_5_N, 150 MHz) *δ*: 204.68 (C-23), 177.94 (C-28), 170.15 (CH_3_COO-), 144.22 (C-13), 122.44 (C-12), 73.50 (C-3), 54.66 (C-4), 51.64 (-OCH_3_), 47.74 (C-9), 47.68 (C-5), 46.90 (C-17), 46.04 (C-19), 42. 01 (C-14), 41.79 (C-18), 39.90 (C-8), 37.72 (C-1), 35.98 (C-10), 35.98 (C-21), 33.94 (C-22), 33.14 (C-20), 32.75 (C-7), 32.22 (C-2), 30.84 (C-29), 28.01 (C-15), 26.13 (C-30), 23.62 (C-27), 23.34 (C-11), 22.81 (C-16), 20.83 (CH_3_COO-), 20.73 (C-6), 17.04 (C-26), 15.48 (C-25), 9.79 (C-24).

#### 4.6.9. Ethyl (3β)-acetyloxy-23-oxoolean-12-en-28-oate (7i)

White solid, yield: 91.6%, mp: 163.5–165.3°C, IR(KBr) νmax: 3430, 2977, 2940, 2876, 2739, 2677, 2604, 2494, 2356, 1731, 1654, 1475, 1444, 1397, 1243, 1172, 1073, 1036, 854, 810, 454 cm^−1^; LC/MS (ESI-MS): *m/z* = 563.2 (M + 23) (positive ion mode); ^1^H NMR (C_5_D_5_N, 600 MHz) *δ*: 9.21 (s, 1H, H-23), 5.23 (br s, 1H, 12-H), 4.91 (dd, *J* = 11.40, 4.80 Hz, 1H, H-3), 4.01 (q, 2H,-OCH_2_CH_3_), 2.80 (dd, *J* = 14.44, 4.63 Hz, 1H, H-18), 1.89 (s, 3H, CH_3_COO-), 1.15 (t, 3H,-OCH_2_ CH_3_), 1.08 (s, 1H, H-24), 1.01 (s, 3H, H-27), 0.91 (s, 3H, H-30), 0.86 (s, 3H, H-26), 0.83 (s, 3H, H-29), 0.68 (s, 3H, H-25); ^13^C NMR (C_5_D_5_N, 150 MHz) *δ*: 204.55 (C-23), 177.63 (C-28), 170.32 (CH_3_COO-), 143.95 (C-13), 121.80 (C-12), 73.38 (C-3), 60.10 (-OCH_2_CH_3_), 54.34 (C-4), 47.84 (C-9), 47.58 (C-5), 46.45 (C-17), 45.88 (C-19), 41.77 (C-14), 41.31 (C-18), 39.68 (C-8), 37.82 (C-1), 35.83 (C-10), 33.88 (C-21), 33.11 (C-22), 32.35 (C-20), 32.07 (C-7), 30.71 (C-29), 29.71 (C-15), 27.60 (C-2), 25.84 (C-11), 23.62 (C-16), 23.36 (C-30), 22.50 (C-27), 21.02 (CH_3_COO-), 20.46 (C-6), 16.97 (C-26), 15.88 (C-25), 14.28 (-OCH_2_CH_3_), 9.49 (C-24).

#### 4.6.10. *n*-Dibutyl (3β)-acetyloxy-23-oxoolean-12-en-28-oate (7j)

White solid, yield: 89.6%, mp: 164.4–166.7°C, IR (KBr) νmax: 3424, 2958, 2926, 2843, 2794, 2679, 1742, 1715, 1468, 1369, 1232, 1205, 1161, 1178, 1117, 1172, 1079, 1024, 969, 936, 816, 756, 619, 553, 465 cm^−1^; LC/MS (ESI-MS): *m/z* = 569.3 (M + 1) (positive ion mode); ^1^H NMR (C_5_D_5_N, 600 MHz) *δ*: 9.52 (s, 1H, H-23), 5.42 (br s, 1H, 12-H), 5.24 (dd, *J* = 11.40, 5.40 Hz, 1H, H-3), 4.19 (q, 2H, -OCH _2_C_3_H_7_), 3.14 (dd, *J* = 13.80, 4.80 Hz, 1H, H-18), 1.94 (s, 3H, CH_3_C OO-), 1.90(dd, 2H, -OCH_2_CH_2_C_2_H_5_), 1.39 (q, 2H, -OC_2_H_4_CH_2_CH_3_), 1.24 (s, 1H, H-24), 1.21 (s, 3H, H-27), 0.98 (s, 3H, H-30), 0.95 (s, 3H, H-26), 0.90 (t, 3H, -OC_3_H_6_CH_3_), 0.90 (s, 3H, H-29), 0.87 (s, 3H, H-25); ^13^C NMR (C_5_D_5_N,150 MHz) *δ*: 204.67 (C-23), 177.42 (C-28), 170.15 (CH_3_COO-), 144.25 (C-13), 122.46 (C-12), 73.50 (C-3), 64.16 (-OCH_2_C_3_H_7_), 54.66 (C-4), 47.74 (C-9), 47.68 (C-5), 46.90 (C-17), 46.09 (C-19), 42.10 (C-14), 41.84 (C-18), 40.00 (C-8), 37.75 (C-1), 35.99 (C-10), 34.01 (C-22), 33.16 (C-20), 32.91 (C-7), 32.36 (C-2), 31.03 (C-21), 30.88 (C-29), 30.02 (-O CH_2_CH_2_C_2_H_5_), 27.98 (C-15), 26.08 (C-30), 23.66 (C-27), 23.32 (C-11), 22.81 (C-16), 20.83 (CH_3_COO-), 20.74 (C-6), 19.55 (-OC_2_H_4_ CH_2_CH_3_), 17.29 (C-26), 15.49 (C-25), 14.28 (-OC_3_H_6_CH_3_), 9.79 (C-24).

### 4.7. General procedure for the synthesis of compounds 8a–8j

NaOH (1.0 ml, 2.0 mol l^−1^) was added to compound **7a** (60 mg, 1.04 × 10^−4^ mol) in C_2_H_5_OH (6 ml). The mixture was allowed to stir for 6 h at room temperature. After completion, the reaction mixture was neutralized with HCl (1.0 mol l^−1^) and organic layer was dried over anhydrous sodium sulfate and evaporated to dryness. The crude material was purified by silica gel chromatography using chloroform–methanol (100 : 3) to obtain compound **8a**. Compounds **8b–8j** were prepared as **8a**.

#### 4.7.1. *N*-Piperidyl-(3β)-hydroxy-23-oxoolean-12-en-28-amide (8a)

White solid, yield: 86.5%, mp: 220.2–223.4°C, IR (KBr) νmax: 3423, 3178, 2932, 2851, 2673, 1728, 1600, 1479, 1421, 1364, 1243, 1134, 1052, 997, 767, 673, 635, 580, 526, 438 cm^−1^; LC/MS (ESI-MS): *m/z* = 538.4 (M + 1) (positive ion mode); ^1^H NMR (C_5_D_5_N, 600 MHz) *δ*: 9.66 (s, 1H, H-23), 5.43 (br s, 1H, 12-H), 4.06 (dd, *J* = 11.10, 5.70 Hz, 1H, H-3), 3.59 (m, 4H, -N(CH_2_)_2_-(CH_2_)_3_), 3.45 (dd, *J*= 15.60, 3.90 Hz, 1H, H-18), 1.50 (m, 4H, -N(CH_2_)_2_-(CH_2_)_2_-CH_2_), 1.40 (s, 1H, H-24), 1.31 (m, 2H, -N(CH_2_)_4_-CH_2_), 1.27 (s, 3H, H-27), 0.98 (s, 3H, H-30), 0.95 (s, 3H, H-26), 0.95 (s, 3H, H-29), 0.89 (s, 3H, H-25); ^13^C NMR (C_5_D_5_N, 150 MHz) *δ*: 207.43 (C-23), 174.49 (C-28), 145.64 (C-13), 121.51 (C-12), 71.73 (C-3), 56.39 (C-4), 48.15 (C-9), 47.84 (C-5), 47.59 (-N(CH_2_)_2_-(CH_2_)_3_), 46.87 (C-17), 46.69 (C-19), 44.27 (C-14), 42.33 (C-18), 39.95 (C-8), 38.54 (C-1), 36.27 (C-10), 36.27 (C-21), 34.34 (C-22), 33.24 (C-20), 32.82 (C-7), 30.63 (C-2), 30.04 (C-29), 28.39 (C-15), 27.13 (C-11), 26.55 (-N(CH_2_)_2_-(CH_2_)_2_-C H_2_), 26.16 (-N(CH_2_)_4_-CH_2_), 25.09 (C-16), 24.22 (C-30), 23.79 (C-27), 21.16 (C-6), 17.34 (C-26), 15.84 (C-25), 9.76 (C-24).

#### 4.7.2. *N*-Morpholino-(3β)-hydroxy-23-oxoolean-12-en-28-amide (8b)

White solid, yield: 84.3%, mp: 223.8–225.1°C, IR (KBr) νmax: 3456, 2946, 2852, 2668, 1729, 1605, 1456, 1417, 1262, 1183, 1114, 1025, 1003, 893, 849, 734, 679, 597, 526 cm^−1^; LC/MS (ESI-MS): *m/z* = 540.5 (M + 1) (positive ion mode); ^1^H NMR (C_5_D_5_N, 600 MHz) *δ*: 9.66 (s, 1H, H-23), 5.46 (br s, 1H, 12-H), 4.10 (dd, *J* = 9.30, 6.90 Hz, 1H, H-3), 3.72–3.80 (m, 4H, -N(CH_2_)_2_(CH_2_)_2_O), 3.67–3.71 (m, 4H, -N(CH_2_)_2_(CH_2_)_2_O), 3.42 (dd, *J* = 14.40, 4.80 Hz, 1H, H-18), 1.40 (s, 1H, H-24), 1.27 (s, 3H, H-27), 0.98 (s, 3H, H-30), 0.96 (s, 3H, H-26), 0.96 (s, 3H, H-29), 0.92 (s, 3H, H-25); ^13^C NMR (C_5_D_5_N, 150 MHz) *δ*: 207.36 (C-23), 174.95 (C-28), 145.34 (C-13), 121.70 (C-12), 71.70 (C-3), 67.18 (-N(CH_2_)_2_(CH_2_)_2_O), 56.35 (C-4), 48.10 (C-9), 47.82 (C-5), 47.58 (-N(CH_2_)_2_(CH_2_)_2_O), 46.66 (C-17), 46.40 (C-19), 44.13 (C-14), 42.30 (C-18), 39.93 (C-8), 38.51 (C-1), 36.23 (C-10), 36.23 (C-21), 34.19 (C-22), 33.18 (C-20), 32.71 (C-7), 30.58 (C-29), 30.08 (C-2), 28.21 (C-15), 27.11 (C-11), 26.16 (C-16), 24.15 (C-30), 23.75 (C-27), 21.13 (C-6), 17.21 (C-26), 15.81 (C-25), 9.73 (C-24).

#### 4.7.3. *N*-(1-Methyl-piperazinyl)-(3β)-hydroxy-23-oxoolean-12-en-28-amide (8c)

White solid, yield: 86.7%, mp: 118.3–189.7°C, IR (KBr) νmax: 3431, 2941, 2859, 2793, 2678, 1731, 1632, 1459, 1417, 1293, 1254, 1141, 1052, 1002, 810, 777, 738, 684, 645, 596, 470 cm^−1^; LC/MS (ESI-MS): *m/z* = 553.4 (M + 1) (positive ion mode); ^1^H NMR (C_5_D_5_N, 600 MHz) *δ*: 9.54 (1H, s, H-23), 5.46 (1H, br s,12-H), 4.10 (1H, dd, *J* = 12.00, 6.00 Hz, H-3), 3.81–3.88 (4H, m, -N(CH_2_)_2_(CH_2_)_2_NCH_3_), 3.43 (1H, dd, *J* = 14.40, 4.20 Hz, H-18), 2.47–2.53 (4H, m, -N(CH _2_)_2_(CH_2_)_2_ N CH_3_), 2.31 (3H, s, -N(CH_2_)_4_NCH_3_), 1.40 (1H, s, H-24), 1.26 (3H, s, H-27), 0.98 (3H, s, H-30), 0.96 (3H, s, H-26), 0.96 (3H, s, H-29), 0.92 (3H, s, H-25); ^13^C NMR (C_5_D_5_N, 150 MHz) *δ*: 207.40 (C-23), 174.86 (C-28), 145.41 (C-13), 121.63 (C-12), 71.71 (C-3), 56.35 (C-4), 55.31 (-N(CH_2_)_2_(CH_2_)_2_NCH_3_), 48.10 (C-9), 47.80 (C-5), 47.59 (C-17), 46.72 (C-19), 45.64 (-N(CH_2_)_4_N-CH_3_), 45.35 (-N(CH _2_)_2_(CH_2_)_2_NCH_3_), 44.15 (C-14), 42.27 (C-18), 39.93 (C-8), 38.50 (C-1), 36.22 (C-10), 36.22 (C-22), 34.24 (C-21), 33.19 (C-20), 32.71 (C-7), 30.57 (C-29), 30.10 (C-2), 28.28 (C-15), 27.09 (C-11), 26.15 (C-16), 24.15 (C-30), 23.76 (C-27), 21.12 (C-6), 17.26 (C-26), 15.81 (C-25), 9.72 (C-24).

#### 4.7.4. *N*-(1-Ethyl-piperazinyl)-(3β)-hydroxy-23-oxoolean-12-en-28-amide (8d)

White solid, yield: 87.2%, mp: 124.7–126.1°C, IR (KBr) νmax: 3442, 2943, 2848, 2815, 2695, 1729, 1604, 1451, 1385, 1353, 1265, 1226, 1089, 1050, 1007, 925, 766, 689, 634, 602, 596, 536 cm^−1^; LC/MS (ESI-MS): *m/z* = 567.5 (M + 1) (positive ion mode); ^1^H NMR (C_5_D_5_N, 600 MHz) *δ*: 9.65(1H, s, H-23), 5.47 (1H, br s, 12-H), 4.10 (1H, t, *J* = 8.00 Hz, H-3), 3.75–3.82 (4H,m,-N(CH_2_)_2_(CH_2_)_2_NC_2_H_5_), 3.44 (1H, d, *J* = 13.8 Hz, H-18), 2.40–2.46 (4H, m, -N(CH_2_)_2_(CH_2_)_2_NC_2_H_5_), 2.33 (2H, q, -N(CH_2_)_4_NCH_2_CH_3_), 1.39 (1H, s, H-24), 1.27 (3H, s, H-27), 1.05 (3H, t, -N(CH_2_)_4_NCH_2_CH_3_), 0.99 (3H, s, H-30), 0.97 (3H, s, H-26), 0.96 (3H, s, H-29), 0.94 (3H, s, H-25); ^13^C NMR (C_5_D_5_N, 150 MHz) *δ*: 207.38 (C-23), 174.74 (C-28), 145.47 (C-13), 121.59 (C-12), 71.70 (C-3), 56.34 (C-4), 53.39 (-N(CH_2_)_2_(CH_2_)_2_N C_2_H_5_), 52.38 (-N(CH_2_)_2_(CH_2_)_2_ NC_2_H_5_), 48.11 (C-9), 47.79 (C-5), 47.57 (C-17), 46.74 (C-19), 45.79 (-N(CH_2_)_4_NCH_2_ CH_3_), 44.16 (C-14), 42.28 (C-18), 39.94 (C-8), 38.50 (C -1), 36.22 (C-10), 36.22 (C-21), 34.25 (C-22), 33.21 (C-20), 32.74 (C-7), 30.58 (C-29), 30.13 (C-2), 28.30 (C-15), 27.07 (C-11), 26.18 (C-16), 24.18 (C-30), 22.82 (C-27), 21.12 (C-6), 17.30 (C-26), 15.80 (C-25), 12.28 (-N(CH _2_)_4_ NCH_2_CH_3_), 9.71 (C-24).

#### 4.7.5. *N*,*N*-Dimethyl-(3β)-hydroxy-23-oxoolean-12-en-28-amide (8e)

White solid, yield: 88.3%, mp: 121.4–123.3°C, IR (KBr) νmax: 3388, 2923, 2852, 2723, 1720, 1605, 1468, 1381, 1265, 1149, 1090, 1052, 975, 904, 823, 739, 690, 613, 569, 531, 465 cm^−1^; LC/MS (ESI-MS): *m/z* = 498.5 (M + 1) (positive ion mode); ^1^H NMR (C_5_D_5_N, 600 MHz) *δ*: 9.65 (s, 1H, H-23), 5.44 (br s, 1H, 12-H), 4.09 (dd, *J* = 10.2, 6.0 Hz, 1H, H-3), 3.47 (dd, *J*= 15.3, 3.9 Hz, 1H, H-18), 3.00 (s, 6H, -N(CH_3_)_2_), 1.39 (s, 1H, H-24), 1.25 (s, 3H, H-27), 0.97 (s, 3H, H-30), 0.95 (s, 3H, H-26), 0.93 (s, 3H, H-29), 0.87 (s, 3H, H-25); ^13^C NMR (C_5_D_5_N, 150 MHz): *δ*: 207.36 (C-23), 175.84 (C-28), 145.50 (C-13), 121.64 (C-12), 71.69 (C-3), 56.36 (C-4), 48.10 (C-9), 47.80 (C-5), 47.59 (C-17), 46.60 (C-19), 43.88 (C-14), 42.32 (C-18), 39.84 (C-8), 38.61 (-N(CH_3_)_2_), 38.48 (C-1), 36.21 (C-10), 34.13 (C-21), 33.25 (C-20), 32.60 (C-7), 30.65 (C-29), 30.02 (C-22), 29.91 (C-2), 28.02 (C-15), 27.10 (C-11), 26.32 (C-16), 24.20 (C-30), 23.74 (C-27), 21.20 (C-6), 17.11 (C-26), 15.75 (C-25), 9.71 (C-24).

#### 4.7.6. *N*,*N*-Diethyl-(3β)-hydroxy-23-oxoolean-12-en-28-amide (8f)

White solid, yield: 87.6%, mp: 121.7–123.5°C, IR (KBr) νmax: 3459, 2926, 2854, 2679, 1732, 1607, 1466, 1416, 1382, 1260, 1205, 1139, 1019, 969, 827, 794, 690, 630, 564, 526, 460 cm^−1^; LC/MS (ESI-MS): *m/z* = 526.6 (M + 1) (positive ion mode); ^1^H NMR (C_5_D_5_N, 600 MHz) *δ*: 9.65 (s, 1H, H-23), 5.47 (br s, 1H, 12-H), 4.09 (dd, *J*= 10.2, 5.7 Hz, 1H, H-3), 3.43 (dd, *J* = 14.45, 4.62 Hz, 1H, H-18), 3.31–3.44 (m, 4H, -N(CH_2_CH_3_)_2_), 1.39 (s, 1H, H-24), 1.27 (s, 3H, H-27), 1.15 (t, 6H, -N(CH_2_CH_3_)_2_), 0.95 (s, 3H, H-30), 0.96 (s, 3H, H-26), 0.96 (s, 3H, H-29), 0.94 (s, 3H, H-25); ^13^C NMR (C_5_D_5_N, 150 MHz) *δ*: 207.34 (C-23), 174.77 (C-28), 145.60 (C-13), 121.42 (C-12), 71.69 (C-3), 56.35 (C-4), 48.09 (C-9), 47.82 (C-5), 47.71 (C-17), 47.12 (C-19), 44.18 (C-14), 42.43 (-N(CH_2_CH_3_)_2_), 42.40 (C-18), 40.04 (C-8), 38.54 (C-1), 36.23 (C-10), 36.23 (C-21), 34.44 (C-22), 33.17 (C-20), 32.86 (C-7), 30.56 (C-29), 30.02 (C-2), 28.48 (C-15), 27.11 (C-11), 25.97 (C-16), 24.14 (C-30), 24.14 (C-27), 21.14 (C-6), 17.56 (C-26), 15.83 (C-25), 13.61 (-N(CH_2_CH_3_)_2_), 9.73 (C-24).

#### 4.7.7. *N*,*N*-(*n*-Dibutyl)-(3β)-hydroxy-23-oxoolean-12-en-28-amide (8g)

White solid, yield: 82.0%, mp: 124.3–125.7°C, IR (KBr) νmax: 3419, 2955, 2926, 1736, 1623, 1465, 1409, 1377, 1249, 1200, 1139, 1106, 734, 663, 593, 526, 476 cm^−1^; LC/MS (ESI-MS): *m/z* = 582.5 (M + 1) (positive ion mode); ^1^H NMR (C_5_D_5_N, 600 MHz) *δ*: 9.65 (s, 1H, H-23), 5.49 (br s, 1H, 12-H), 4.10 (dd, *J* = 11.4, 5.7 Hz, 1H, H-3), 3.46 (dd, *J* = 14.46, 4.62 Hz, 1H, H-18), 3.40 (br s, 4H, -N(CH_2_-C_3_H _7_)_2_), 1.39 (s, 1H, H-24), 1.31–1.36 (m, 8H, -N(CH_2_C_2_H_4_CH_3_)_2_), 1.30 (s, 3H, H-27), 1.00 (s, 3H, H-30), 0.98 (s, 3H, H-26), 0.96 (s, 3H, H-29), 0.95 (s, 3H, H-25), 0.95 (t, 6H, -N(C_3_H_6_CH_3_)_2_); ^13^C NMR (C_5_D_5_N, 150 MHz) *δ*: 207.35 (C-23), 174.99 (C-28), 145.68 (C-13), 121.47 (C-12), 71.70 (C-3), 56.35 (C-4), 48.43 (-N(CH_2_C_3_H_7_)_2_), 48.11 (C-9), 47.92 (C-5), 47.81 (C-17), 47.21 (C-19), 44.3 (C-14), 42.46 (C-18), 40.08 (C-8), 38.54 (C-1), 36.24 (C-10), 36.24 (C-21), 34.43 (C-22), 33.16 (C-20), 32.88 (C-7), 30.58 (C-29), 30.01 (C-2), 28.59 (C-15), 27.11 (C-11), 26.03 (-N(CH_2_CH_2_C_2_H_5_)_2_), 24.23 (C-16), 23.80 (C-30), 23.01 (-N(C_2_H_4_CH_2_CH_3_)_2_), 21.13 (C-27), 20.71 (C-6), 14.20 (-N(C_3_H_6_CH_3_)_2_), 17.61(C-26), 15.82(C-25), 9.73 (C-24).

### 4.8. General procedure for the synthesis of compounds 9a–9j

Oxalyl chloride (2.0 × 10^−1^ ml, 2.36 × 10^−3^ mol) was added to compound **6** (46 mg, 7.8 × 10^−5^ mol) in CH_2_Cl_2_ (3 ml). The mixture was allowed to stir for 12 h at room temperature. After completion, the reaction mixture was neutralized with Et_3_N and evaporated to dryness. To a stirred solution of the mixture in dry CH_2_Cl_2_ (3 ml) was added piperidine (4.0 × 10^−2^ ml, 4.04 × 10^−4^ mol). The stirring was continued for 8 h at room temperature. After CH_2_Cl_2_ evaporation, water (5 ml) was added to this mixture, and the mixture was extracted with ethyl acetate (3 × 5 ml). The combined organic layer was dried over anhydrous sodium sulfate and evaporated to dryness. The crude material was purified by silica gel chromatography using petroleum ether–ethyl acetate (1 : 1) to afford compound **9a**. Compounds **9b–9j** were prepared as **9a**.

#### 4.8.1. *N*-Piperidyl-(3β)-acetyloxy-23-[(2,4-dinitrophenyl) hydrazono]olean-12-en-28-amide (9a)

Yellow solid, yield: 92.4%, mp: 117.2–118.1°C, IR(KBr) νmax: 3441, 3283, 3099, 2928, 2854, 2036, 1726, 1618, 1590, 1520, 1465, 1423, 1332, 1308, 1244, 1136, 1030, 951, 914, 825, 741, 599, 530, 441 cm^−1^; LC/MS (ESI-MS): *m/z* = 760.6 (M + 1) (positive ion mode); ^1^H NMR (CDCl_3_, 600 MHz) *δ*: 10.99 (s, 1H, -CHNNH-), 9.11 (d, 1H, 2.4 Hz,PhH-3), 8.30 (dd, 9.6, 2.4 Hz, 1H, PhH-5), 7.90 (d, 9.6 Hz, 1H, PhH-6), 7.25 (s, 1H, -CHNNH-), 5.28 (brs, 1H, 12-H), 4.90 (dd, *J* = 11.40, 4.80 Hz, 1H, H-3), 3.52–3.57 (m, 4H, -N(CH_2_)_2_(CH_2_)_3_), 3.10 (dd, *J* = 14.10, 4.50 Hz, 1H, H-18), 1.94 (s, 3H, CH_3_ COO-), 1.46–1.51 (m, 4H, -N(CH_2_)_2_(CH_2_)_2_ CH_2_), 1.30–1.36 (m, 2H, -N(CH_2_)_4_CH_2_), 1.23 (s, 1H, H-24), 1.16 (s, 3H, H-27), 1.04 (s, 3H, H-30), 0.93 (s, 3H, H-26), 0.90 (s, 3H, H-29), 0.78 (s, 3H, H-25); ^13^C NMR (CDCl_3_, 150 MHz) *δ*: 174.64 (C-28), 170.52 (CH_3_COO-), 158.78 (C-23), 145.15 (Ph-1), 145.14 (C-13), 137.79 (Ph-4), 129.99 (Ph-5), 128.93 (Ph-2), 123.50 (Ph-6), 120.81 (C-12), 116.44 (Ph-3), 76.06 (C-3), 58.45, 53.44 (-N(CH_2_)_2_(CH_2_)), 52.20 (C-4), 48.02 (C-17), 47.34 (C-9), 47.30 (C-5), 46.56 (C-19), 43.66 (C-14), 41.92 (C-18), 39.44 (C-8), 37.86 (C-1), 36.59 (C-10), 36.59 (C-21), 34.09 (C-22), 33.09 (C-20), 32.49 (C-7), 30.40 (C-29), 29.70 (C-2), 28.01 (C-15), 26.16 (C-30), 25.91, 24.10 (-N(CH_2_)_2_ (CH_2_)_2_CH_2_), 24.82 (C-11), 23.35 (C-16), 22.96 (C-27), 21.21 (CH_3_COO-), 20.19 (C-6), 18.45 (-N(CH_2_)_4_CH_2_), 16.94 (C-26), 15.84 (C-25), 11.99 (C-24).

#### 4.8.2. *N*-Morpholino-(3β)-acetyloxy-23-[(2,4-dinitrophenyl) hydrazono]olean-12-en-28-amide (9b)

Yellow solid, yield: 85%; mp: 114.2–116.1°C, IR(KBr) νmax: 3293, 3112, 2924, 2853, 1727, 1619, 1591, 1521, 1456, 1425, 1378, 1332, 1246, 1120, 1026, 1004, 946, 915, 832, 734, 613, 520 cm^−1^; LC/MS (ESI-MS): *m/z* = 762.6 (M + 1) (positive ion mode); ^1^H NMR (CDCl_3_, 600 MHz) *δ*: 10.99 (s, 1H,-CHNNH-), 9.12 (d,3.0 Hz, 1H, PhH-3), 8.30 (dd, 9.6, 2.4 Hz, 1H, PhH-5), 7.90 (d, 9.6 Hz, 1H, PhH-6), 7.23 (s, 1H, -CHNNH-), 5.29 (br s, 1H, 12-H), 4.90 (dd, *J* = 11.40, 4.80 Hz, 1H, H-3), 3.59–3.69 (m, 8H, -N(CH_2_)_4_O), 3.09 (dd, *J* = 15.00, 4.20 Hz, 1H, H-18), 1.94 (s, 3H, CH_3_COO-), 1.23 (s, 1H, H-24), 1.17 (s, 3H, H-27), 1.03 (s, 3H, H-30), 0.94 (s, 3H, H-26), 0.91 (s, 3H, H-29), 0.76 (s, 3H, H-25); ^13^C NMR (CDCl_3_, 150 MHz) *δ*: 175.10 (C-28), 170.52 (CH_3_COO-), 158.67 (C-23), 145.14 (Ph-1), 144.77 (C-13), 137.82 (Ph-4), 130.00 (Ph-5), 128.95 (Ph-2), 123.50 (Ph-6), 121.13 (C-12), 116.44 (Ph-3), 76.02 (C-3), 66.95 (-N(CH _2_)_2_(CH_2_)_2_O), 52.20 (C-4), 47.98 (C-9), 47.98 (C-5), 47.40 (-N(CH 2)2(CH2)2O), 47.29 (C-17), 46.35 (C-19), 43.57 (C-14), 41.92 (C-18), 39.46 (C-8), 37.86 (C-1), 36.58 (C-10), 33.96 (C-22), 33.04 (C-20), 32.45 (C-7), 31.93 (C-21), 30.39 (C-29), 27.86 (C-2), 25.95 (C-15), 24.04 (C-11), 23.34 (C-30), 22.95 (C-27), 22.73 (C-16), 21.21 (CH_3_COO-), 20.16 (C-6), 16.90 (C-26), 15.83 (C-25), 11.99 (C-24).

#### 4.8.3. *N*-(1-Methyl-piperazinyl)-(3β)-acetyloxy-23-[(2,4-dinitrophenyl) hydrazono] olean-12-en-28-amide (9c)

Yellow solid, yield: 85%, mp: 115.3–117.1°C, IR (KBr) νmax : 3446, 3298, 3112, 2927, 2853, 2778, 1732, 1618, 1591, 1519, 1458, 1425, 1371, 1332, 1308, 1243, 1139, 1027, 915, 832, 739, 602, 539, 493 cm^−1^; LC/MS (ESI-MS): *m/z* = 775.8 (M + 1) (positive ion mode); ^1^H NMR (CDCl_3_, 600 MHz) *δ*: 11.00 (s, 1H, CHNNH-), 9.12 (d, 2.4 Hz, 1H, PhH-3), 8.30 (dd, 9.6, 2.4 Hz, 1H, PhH-5), 7.90 (d, 9.6 Hz, 1H, PhH-6), 7.24 (s, 1H, -CHNNH-), 5.28 (br s, 1H, 12-H), 4.90 (dd, *J* = 11.40, 4.20 Hz, 1H, H-3), 3.61–3.75 (m, 4H, -N(CH_2_)_2_ (CH_2_)_2_NCH_3_), 3.08 (dd, *J* = 14.40, 4.20 Hz, 1H, H-18), 2.38–2.46 (m, 4H, -N(CH_2_)_2_(CH_2_)_2_NCH_3_), 2.32 (s, 3H, -N(CH_2_)_4_NCH_3_), 1.95 (s, 3H, CH_3_COO-), 1.23 (s, 1H, H-24), 1.16 (s, 3H, H-27), 1.03 (s, 3H, H-30), 0.93 (s, 3H, H-26), 0.90 (s, 3H, H-29), 0.76 (s, 3H, H-25); ^13^C NMR (CDCl_3_, 150 MHz) *δ*: 174.97 (C-28), 170.53 (CH_3_COO-), 158.72 (C-23), 145.15 (Ph-1), 144.86 (C-13), 137.80 (Ph-4), 130.00 (Ph-5), 128.93 (Ph-2), 123.50 (Ph-6), 122.05 (C-12), 116.44 (Ph-3), 76.06 (C-3), 55.09 (-N(CH_2_)_2_(CH_2_)_2_NCH_3_), 52.18 (C-4), 47.99 (-N (CH_2_)_2_(CH_2_)_2_NCH_3_), 47.39 (C-9), 47.29 (C-5), 46.39 (C-17), 45.81 (C-19), 45.04 (-N(CH_2_)_4_NCH_3_), 43.58 (C-14), 41.90 (C-18), 39.45 (C-8), 37.86 (C-1), 36.58 (C-10), 34.00 (C-22), 33.05 (C-20), 32.45 (C-7), 30.39 (C-29), 29.93 (C-21), 29.70 (C-2), 27.90 (C-15), 22.94 (C-11), 24.05 (C-16), 23.34 (C-30), 22.95 (C-27), 21.21 (CH_3_COO-), 20.18 (C-6), 16.92 (C-26), 15.84 (C-25),11.99 (C-24).

#### 4.8.4. *N*-(1-Ethyl-piperazinyl)-(3β)-acetyloxy-23-[(2,4-dinitrophenyl) hydrazono] olean-12-en-28-amide (9d)

Yellow solid, yield: 85%, mp: 124.4–126.5°C, IR (KBr) νmax 3446, 3293, 3101, 2923, 2852, 1732, 1618, 1590, 1519, 1463, 1376, 1332, 1308, 1240, 1068, 1024, 991, 920, 832, 739, 635, 536, 460 cm^−1^; LC/MS (ESI-MS): *m/z* = 789.8 (M + 1) (positive ion mode); ^1^H NMR (CDCl_3_, 600 MHz) *δ*: 11.00 (s, 1H, -CHNNH-), 9.11 (d, 2.4 Hz, 1H, PhH-3), 8.30 (dd, 9.6, 2.4 Hz, 1H, PhH-5),7.90 (d, 9.6 Hz, 1H, PhH-6), 7.24 (s, 1H, -CHNNH-), 5.28 (br s, 1H, 12-H), 4.90 (dd, *J* = 11.70, 4.50 Hz, 1H, H-3), 3.64–3.74 (m, 4H, -N(CH_2_)_2_(CH_2_)_2_NC_2_ H_5_), 3.09 (dd, *J* = 14.40, 4.20 Hz, 1H, H-18), 2.40–2.48 (m, 4H, -N (CH_2_)_2_ (CH_2_)_2_NC_2_H_5_), 2.45 (q, 2H, -N(CH_2_)_4_NCH_2_CH_3_), 1.94 (s, 3H, CH_3_COO-), 1.23 (s, 1H, H-24), 1.16 (s, 3H, H-27), 1.11 (t, 3H, -N(CH_2_)_4_ NCH_2_CH_3_), 1.03 (s, 3H, H-30), 0.93 (s, 3H, H-26), 0.92 (s, 3H, H-29), 0.76 (s, 3H, H-25); ^13^C NMR (CDCl_3_, 150 MHz) *δ*: 174.90 (C-28), 170.51 (CH_3_COO-), 158.72 (C-23), 145.14 (Ph-1), 144.89 (C-13), 137.80 (Ph-4), 129.99 (Ph-5), 128.93 (Ph-2), 123.49 (Ph-6), 122.03 (C-12), 116.44 (Ph-3), 76.06 (C-3), 52.90 (-N(CH_2_)_2_ (CH_2_)_2_NC_2_ H_5_), 52.30 (-N(CH_2_)_2_(CH_2_)_2_NC_2_H_5_), 52.17 (C-4), 47.99 (C-9), 47.38 (C-5), 47.29 (C-17), 46.39 (C-19), 45.08 (-N(C H_2_)_4_ NCH_2_CH_3_), 43.57 (C-14), 41.90 (C-18), 39.45 (C-8), 37.86 (C-1), 36.58 (C-10), 34.00 (C-22), 33.06 (C-20), 32.45 (C-7), 31.93 (C-21), 30.39 (C-29), 29.36 (C-2), 27.90 (C-15), 24.16 (C-11), 23.34 (C-16), 22.95 (C-30), 22.69 (C-27), 21.20 (CH_3_COO-), 20.18 (C-6), 16.94 (C-26), 15.83 (C-25), 14.13 (-N(CH_2_)_4_NCH_2_CH_3_), 11.98 (C-24).

#### 4.8.5. *N*,*N*-Dimethyl-(3β)-acetyloxy-23-[(2,4-dinitrophenyl) hydrazono]olean-12-en-28-amide (9e)

Yellow solid, yield: 85%, mp: 121.5–123.2°C, IR (KBr) νmax: 3446, 3298, 3101, 2928, 2854, 1732, 1618, 1590, 1519, 1464, 1428, 1371, 1332, 1308, 1242, 1139, 1079, 1028, 958, 926, 827, 734, 641, 526, 432 cm^−1^; LC/MS (ESI-MS): *m/z* = 720.4 (M + 1) (positive ion mode); ^1^H NMR (CDCl_3_, 600 MHz) *δ*: 10.99 (s, 1H, -CHNNH-), 9.12 (d, 3.0 Hz, 1H, PhH-3), 8.30 (dd, 9.3, 2.7 Hz, 1H, PhH-5), 7.90 (d, 9.6 Hz, 1H, PhH-6), 7.24 (s, 1H, -CHNNH-), 5.28 (br s, 1H, 12-H), 4.90 (dd, *J* = 11.70, 4.50 Hz, 1H, H-3), 3.15 (m, 1H, H-18), 3.01 (s, 6H, -N(CH_3_)_2_), 1.94 (s, 3H, CH_3_COO-),1.23 (s, 1H, H-24), 1.16 (s, 3H, H-27), 1.03 (s, 3H, H-30), 0.94 (s, 3H, H-26), 0.90 (s, 3H, H-29), 0.75 (s, 3H, H-25); ^13^C NMR (CDCl_3_, 150 MHz) *δ*: 176.21 (C-28), 170.53 (CH_3_COO-), 158.76 (C-23), 145.15 (Ph-1), 144.99 (C-13), 137.80 (Ph-4), 130.00 (Ph-5), 128.94 (Ph-2), 123.50 (Ph-6), 121.08 (C-12), 116.45 (Ph-3), 76.05 (C-3), 52.20 (C-4), 48.01 (C-9), 47.42 (C-5), 47.30 (C-17), 46.31 (C-19), 43.33 (C-14), 41.96 (C-18), 39.39 (C-8), 38.72 (-N(CH_3_)_2_), 37.84 (C-1), 36.58 (C-10), 33.93 (C-22), 33.13 (C-20), 32.37 (C-7), 30.48 (C-29), 29.70 (C-2), 29.63 (C-21), 27.69 (C-15), 26.10 (C-11), 24.09 (C-16), 23.35 (C-30), 22.96 (C-27), 21.21 (CH_3_COO-), 20.18 (C-6), 16.84 (C-26), 15.80 (C-25), 11.97 (C-24).

#### 4.8.6. *N*,*N*-Diethyl-(3β)-acetyloxy-23-[(2,4-dinitrophenyl) hydrazono]olean-12-en-28-amide (9f)

Yellow solid, yield: 85%, mp: 119.7–121.2°C, IR (KBr) νmax: 3479, 3292, 3101, 2949, 2849, 1726, 1619, 1520, 1468, 1423, 1332, 1307, 1246, 1137, 1069, 1029, 963, 908, 837, 744, 601, 530, 448 cm^−1^; LC/MS (ESI-MS): *m/z* = 748.6 (M + 1) (positive ion mode); ^1^H NMR (CDCl_3_, 600 MHz) *δ*: 10.99 (s, 1H, -CHNNH-), 9.11 (d, 3.0 Hz, 1H, PhH-3), 8.30 (dd, 9.6, 2.4 Hz, 1H, PhH-5), 7.90 (d, 9.6 Hz, 1H, PhH-6), 7.24 (s, 1H, -CHNNH-), 5.28 (br s, 1H, 12-H), 4.90 (dd, *J* = 11.40, 4.20 Hz, 1H, H-3), 3.26–3.33 (m, 4H, -N(CH_2_CH_3_)_2_), 3.09 (dd, *J* = 14.40, 4.2 Hz, 1H, H-18), 1.94 (s, 3H, CH_3_COO-), 1.23 (s, 1H, H-24), 1.17 (s, 3H, H-27), 1.11–1.14 (m, 6H, -N(CH_2_CH_3_)_2_), 1.04 (s, 3H, H-30), 0.93 (s, 3H, H-26), 0.90 (s, 3H, H-29), 0.80 (s, 3H, H-25); ^13^C NMR (CDCl_3_, 150 MHz) *δ*: 174.85 (C-28), 170.53 (CH_3_COO-), 158.78 (C-23), 145.15 (Ph-1), 145.11 (C-13), 137.79 (Ph-4), 129.99 (Ph-5), 128.92 (Ph-2), 123.50 (Ph-6), 120.77 (C-12), 116.44 (Ph-3), 76.06 (C-3), 52.20 (C-4), 48.00 (C-9), 47.48 (C-5), 47.30 (C-17), 46.80 (C-19), 43.67 (C-14), 42.14 (-N(CH_2_CH_3_)_2_), 42.01 (C-18), 39.54 (C-8), 37.89 (C-1), 36.59 (C-10), 36.59 (C-21), 34.24 (C-22), 33.05 (C-20), 32.57 (C-7), 30.37 (C-29), 29.70 (C-2), 28.12 (C-15), 25.77 (C-11), 24.07 (C-16), 23.35 (C-30), 22.52 (C-27), 21.21 (CH_3_ COO-), 20.19 (C-6), 17.16 (C-26), 15.88 (C-25), 13.31 (-N(CH_2_CH _3_)_2_), 11.99 (C-24).

#### 4.8.7. *N*,*N*-(*n*-Dibutyl)-(3β)-acetyloxy-23-[(2,4-dinitrophenyl) hydrazono]olean-12-en-28-amide (9g)

Yellow solid, yield: 85%, mp: 112.6–113.9°C, IR (KBr) νmax: 3293, 3112, 2954, 2871, 1733, 1619, 1592, 1520, 1465, 1426, 1332, 1308, 1241, 1139, 1068, 1030, 958, 920, 832, 745, 641, 536, 443 cm^−1^; LC/MS (ESI-MS): *m/z* = 803.0 (M − 1) (negative ion mode); ^1^H NMR (CDCl_3_, 600 MHz) *δ*: 10.99 (s, 1H, -CHNNH-),9.12 (d, 3.0 Hz, 1H, PhH-3), 8.30 (dd, 9.6, 2.4 Hz, 1H, PhH-5), 7.90 (d, 9.0 Hz, 1H, PhH-6), 7.24 (s, 1H, -CHNNH-), 5.28 (br s, 1H, 12-H), 4.90 (dd, *J* = 11.40, 4.80 Hz, 1H, H-3), 3.07–3.17 (m, 4H, -N(CH_2_C_3_H_7_)_2_), 3.08 (d, *J* = 13.8 Hz, 1H, H-18), 1.94 (s, 3H, CH_3_COO-), 1.48–1.51 (m, 4H, -N (CH_2_CH_2_C_2_H_5_)_2_), 1.27–1.29 (m, 4H, -N(C_2_H_4_CH_2_CH_3_)_2_), 1.23 (s, 1H, H-24), 1.16 (s, 3H, H-27), 1.04 (s, 3H, H-30), 0.93 (s, 3H, H-26), 0.92 (t, 6H, -N(C_3_H_6_CH_3_)_2_), 0.90 (s, 3H, H-29), 0.79 (s, 3H, H-25); ^13^C NMR (C_5_D_5_N, 150 MHz) *δ*: 174.98 (C-28), 170.52 (CH_3_COO-), 158.79 (C-23), 145.16 (Ph-1), 145.16 (C-13), 137.79 (Ph-4), 129.99 (Ph-5), 128.93 (Ph-2), 123.50 (Ph-6), 120.77 (C-12), 116.44 (Ph-3), 76.09 (C-3), 52.18 (C-4), 48.01 (C-9), 48.01 (C-5), 47.60 (C-17), 47.30 (-N(CH_2_C_3_H_7_)_2_), 46.89 (C-19), 43.67 (C-14), 42.03 (C-18), 39.54 (C-8), 37.89 (C-1), 36.60 (C-10), 36.60 (C-21), 34.25 (C-22), 33.03 (C-20), 32.56 (C-7), 30.38 (C-29), 29.70 (C-2), 28.18 (C-15), 25.76 (C-11), 24.10 (C-16), 23.34 (C-30), 22.97 (C-27), 21.21 (CH_3_ COO-), 20.42 (-N(CH_2_CH_2_C_2_H_5_)_2_), 20.19 (C-6), 18.45 (-N(C_2_H_4_C H_2_CH_3_)_2_), 17.16 (C-26), 15.89 (C-25), 13.98 (-N(C_3_H_6_CH_3_)_2_), 12.00 (C-24).

#### 4.8.8. Methyl (3β)-acetyloxy-23-[(2,4-dinitrophenyl)hydrazono] olean-12-en-28-oate (9h)

Yellow solid, yield: 85%, mp: 134.4–136.1°C, IR (KBr) νmax: 3287, 3106, 2936, 2860, 2853, 1736, 1616, 1583, 1523, 1518, 1463, 1435, 1336, 1232, 1079, 1030, 1029, 969, 920, 832, 739, 613, 515, 449 cm^−1^; LC/MS (ESI-MS): *m/z* = 705.7 (M − 1) (negative ion mode); ^1^H NMR (CDCl_3_, 600 MHz) *δ*: 10.99 (s, 1H, -CHNNH-), 9.12 (d, 2.4 Hz, 1H, PhH-3), 8.30 (dd, 9.6, 2.4 Hz, 1H, PhH-5), 7.90 (d, 9.6 Hz, 1H, PhH-6), 7.24 (s, 1H, -CHNNH-), 5.30 (br s, 1H, 12-H), 4.90 (dd, *J* = 11.70, 4.50 Hz, 1H, H-3), 3.62 (q, 3H, -OCH_3_), 2.87 (dd, *J* = 14.10, 4.50 Hz, 1H, H-18), 1.95 (s, 3H, CH_3_COO-), 1.24 (s, 1H, H-24), 1.16 (s, 3H, H-27), 1.04 (s, 3H, H-30), 0.93 (s, 3H, H-26), 0.90 (s, 3H, H-29), 0.75 (s, 3H, H-25); ^13^C NMR (CDCl_3_,150 MHz) *δ*: 178.22 (C-28), 170.53 (CH_3_COO-), 158.62 (C-23), 145.15 (Ph-1), 143.89 (C-13), 137.82 (Ph-4), 130.00 (Ph-5), 128.96 (Ph-2), 123.51 (Ph-6), 121.91 (C-12), 116.44 (Ph-3), 76.04 (C-3), 52.10 (C-4), 51.56 (-OCH_3_), 47.83 (C-9), 47.27 (C-5), 46.69 (C-17), 45.84 (C-19), 41.70 (C-14), 41.30 (C-18), 39.61 (C-8), 37.89 (C-1), 36.52 (C-10), 33.82 (C-22), 33.10 (C-20), 32.31 (C-7), 32.25 (C-21), 30.70 (C-29), 29.70 (C-2), 27.68 (C-15), 25.93 (C-30), 22.63 (C-27), 23.37 (C-11), 23.00 (C-16), 21.21 (CH_3_COO-), 20.15 (C-6), 16.85 (C-26), 15.80 (C-25), 12.01 (C-24).

#### 4.8.9. Ethyl(3β)-acetyloxy-23-[(2,4-dinitrophenyl)hydrazono] olean-12-en-28-oate (9i)

Yellow solid, yield: 85%, mp: 135.5–136.8°C, IR (KBr) νmax: 3291, 2977, 2924, 2853, 1739, 1719, 1621, 1591, 1518, 1464, 1420, 1369, 1338, 1250, 1135, 1029, 958, 920, 838, 745, 602, 536 cm^−1^; LC/MS (ESI-MS): *m/z* = 720.2 (M − 1) (negative ion mode); ^1^H NMR (CDCl_3_, 600 MHz) *δ*: 10.99 (s, 1H, -CHNNH-), 9.12 (d, 2.4 Hz, 1H, PhH-3), 8.30 (dd, 9.3, 2.7 Hz, 1H, PhH-5), 7.90 (d, 9.6 Hz, 1H, PhH-6), 7.23 (s, 1H, -CHNNH-), 5.30 (br s, 1H, 12-H), 4.90 (dd, *J* = 11.70, 4.50 Hz, 1H, H-3), 4.08 (q, 2H, -OCH_2_CH_3_), 2.88 (dd, *J* = 14.44, 4.63 Hz, 1H, H-18), 1.94 (s, 3H, CH_3_COO-), 1.23 (s, 1H, H-24), 1.22 (t, 3H, -OCH_2_CH_3_), 1.16 (s, 3H, H-27), 1.04 (s, 3H, H-30), 0.93 (s, 3H, H-26), 0.90 (s, 3H, H-29), 0.77 (s, 3H, H-25); ^13^C NMR (CDCl_3_, 150 MHz) *δ*: 177.64 (C-28), 170.52 (CH_3_COO-), 158.60 (C-23), 145.13 (Ph-1), 143.90 (C-13), 137.82 (Ph-4), 129.99 (Ph-5), 128.95 (Ph-2), 123.50 (Ph-6), 121.83 (C-12), 116.42 (Ph-3), 76.03 (C-3), 60.10 (-OCH_2_ CH_3_), 52.10 (C-4), 47.82 (C-9), 47.27 (C-5), 46.46 (C-17), 45.88 (C-19), 41.76 (C-14), 41.30 (C-18), 39.67 (C-8), 37.90 (C-1), 36.51 (C-10), 36.51 (C-21), 33.87 (C-22), 33.10 (C-20), 32.34 (C-7), 30.71 (C-29), 29.66 (C-2), 27.63 (C-15), 25.84 (C-11), 23.61 (C-16), 23.38 (C-30), 22.93 (C-27), 21.21 (CH_3_COO-), 20.14 (C-6), 16.98 (C-26), 15.81 (C-25), 14.27 (-OCH_2_CH_3_), 12.01 (C-24).

#### 4.8.10. *n*-Butyl(3β)-acetyloxy-23-[(2,4-dinitrophenyl) hydrazono]olean-12-en-28-oate (9j)

Yellow solid, yield: 85%, mp: 137.6–139.3°C; IR (KBr) νmax: 3298, 3106, 2951, 2872, 1728, 1618, 1591, 1519, 1463, 1426, 1366, 1333, 1240, 1139, 1071, 1029, 969, 909, 827, 739, 646, 536, 454 cm^−1^; LC/MS (ESI-MS): *m/z* = 747.8 (M − 1) (negative ion mode); ^1^H NMR (CDCl_3_, 600 MHz) *δ*: 10.99 (s, 1H, -CHNNH-), 9.12 (d, 2.4 Hz, 1H, PhH-3), 8.30 (dd, 9.6, 2.4 Hz, 1H, PhH-5), 7.90 (d, 9.6 Hz, 1H, PhH-6), 7.23 (s, 1H, -CHNNH-), 5.30 (br s, 1H, 12-H), 4.90 (dd, *J* = 11.40, 4.80 Hz, 1H, H-3), 4.01 (t, 2H, -OCH_2_C_3_H_7_), 2.88 (dd, *J* = 13.80, 4.20 Hz, 1H, H-18), 1.95 (s, 3H, CH_3_COO-), 1.92 (q, 2H, -O CH_2_CH_2_C_2_H_5_), 1.38 (q, 2H, -OC_2_H_4_CH_2_CH_3_), 1.23 (s, 1H, H-24), 1.16 (s, 3H, H-27), 1.04 (s, 3H, H-30), 0.93 (s, 3H, H-26), 0.93 (t, 3H, -OC_3_H_6_CH_3_), 0.90 (s, 3H, H-29), 0.76 (s, 3H, H-25); ^13^C NMR (CDCl_3_, 150 MHz) *δ*: 177.70 (C-28), 170.53 (CH_3_COO-), 158.62 (C-23), 145.15 (Ph-1), 143.93 (C-13), 137.82 (Ph-4), 130.00 (Ph-5), 128.96 (Ph-2), 123.51 (Ph-6), 121.89 (C-12), 116.44 (Ph-3), 76.04 (C-3), 64.00 (-OCH_2_C_3_H_7_), 52.09 (C-4), 47.82 (C-9), 47.28 (C-5), 46.63 (C-17), 45.87 (C-19), 41.77 (C-14), 41.34 (C-18), 39.66 (C-8), 37. 91 (C-1), 36.52 (C-10), 33.87 (C-22), 33.11 (C-20), 32.40 (C-7), 32.35 (C-21), 30.72 (C-29), 30.69 (-OCH_2_CH_2_C_2_H_5_), 29.71 (C-2), 27.63 (C-15), 25.87 (C-30), 23.61 (C-27), 23.39 (C-11), 27.97 (C-16), 21.21 (CH_3_COO-), 20.16 (C-6), 19.25 (-OC_2_H_4_CH_2_CH_3_), 17.01 (C-26), 15.81 (C-25), 13.73 (-OC_3_H_6_CH_3_), 12.01 (C-24).

### 4.9. Biology

A549, LOVO, SKOV3 and HepG2 cell lines were purchased from American Type Culture Collection (ATCC). Trypsin, EDTA and fetal bovine serum (FBS) were purchased from Sigma Chemicals Co. (St. Louis, MO). AO and EB were purchased from KeyGEN BioTECH Co. (Nanjing, China). PI, RNase A, RPMI-1640 Hyclone, phosphate buffered solution (PBS), MTT and 6- and 96-well flat bottom tissue culture plates were purchased from Beyotime Biotechnology Co. (Shanghai, China).

#### 4.9.1. Cytotoxicity screening using MTT assay

MTT assay is a standard colorimetric assay for measuring cellular proliferation, in which MTT is taken up by living cells and reduced by a mitochondrial dehydrogenase enzyme to a purple formazan product that is impermeable to the cell membrane. Solubilization with solvents like dimethylsulfoxide (DMSO) leads to liberation of product and amount of purple formazan product is directly related to the cell viability. Cells were seeded in 96-well plates at a density of 1.0 × 10^5^ cells ml^−1^ and incubated with increasing concentrations of agents (corresponding to 100, 10, 1, 0.1, and 0.01 µM of the compounds or controls) for 48 h at 37°C in RPMI-1640 with 10% FBS medium. Then the above media were replaced with 90 µl of fresh serum free media and 10 µl of MTT reagent (5 mg ml^−1^) and plates were incubated at 37°C for 4 h. Then the above media were replaced with 150 ml of DMSO and incubated at 37°C for 20 min. The absorbance at 570 nm was recorded using Thermo Scientific SkanIt software. The IC_50_ values were analysed using IBM SPSS 22.0 software.

#### 4.9.2. Morphological observation

Cellular morphological effects of compounds **4** and **7g** were determined by the AO/EB double-staining method [24]. Cells were seeded in 6-well plates at a concentration of 1 × 10^6^ cells ml^−1^ for 24 h at 37°C in RPMI-1640 with 10% FBS medium. After incubation, cells were treated with different concentrations of compounds **4** and **7 g** for 48 h. Then the cells were collected and washed two times by PBS. Cells were seeded in 48-well plates at a concentration of 4 × 10^6^ cells ml^−1^. 10 µl of fluorescent dyes containing AO and EB added into each well in equal volumes (10 mg ml^−1^) respectively and within 10 min the cells were visualized under an inverted fluorescence microscope (Nikon ECLIPSE Ti, Japan) with a blue filter at 200× magnification [27]. At least 200 cells were randomly counted in various fields. The tests were repeated three times. The percentage of apoptotic cells was calculated by the following formula: apoptotic rate (%) = (numbers of early apoptotic cells + numbers of late apoptotic cells)/(numbers of all the cells counted).

#### 4.9.3. Cell cycle analysis

To determine the effect of compounds **4** and **7g** on the cell cycle, cells were seeded in 6-well plates at a concentration of 1 × 10^5^ cells ml^−1^ for 24 h at 37°C in RPMI-1640 with 10% FBS medium. After incubation, cells were treated with different concentrations of compounds **4** and **7 g** for 48 h. Then the cells were collected, washed and fixed in 70% ethanol at 4°C. After 12 h, fixed cells were pelleted and stained with cell cycle analysis reagent as per the manufacturer instructions for 30 min at 37°C in the dark. The data were recorded by a COULTER® EPICS® XL™ flow cytometer (Beckman Coulter, USA). The results were analysed by Expo 32 ADC software.

## Data accessibility

Further details are available in the electronic supplementary material. This includes further experimental data and various NMR, UV and MS spectra.

## Authors' contributions

Jingyong Sun, F.W. and Y.M. contributed to the conception of the study. Jie Sun contributed significantly to analysis and manuscript preparation. H.Z. and L.S. performed the data analyses and wrote the manuscript. Y.L. helped perform the analysis with constructive discussions.

## Competing interests

We declare we have no competing interests.

## Funding

The authors for correspondence are grateful to support from the Project of Shandong Province Higher Educational Science and Technology Program (J15LM07) and the Innovation Project of Shandong Academy of Medical Sciences.

## Supplementary Material

As paper for “Synthesis of gypsogenin derivatives with capabilities to arrest cell cycle and induce apoptosis in human cancer cells”
