# Supplementary material for: Synthesis of gypsogenin derivatives with capabilities to arrest cell cycle and induce apoptosis in human cancer cells
Source: R Soc Open Sci. 2018 Jan 24;5(1):171510. doi: 10.1098/rsos.171510 (PMC5792931; doi:10.1098/rsos.171510)
Supplement: As paper for “Synthesis of gypsogenin derivatives with capabilities to arrest cell cycle and induce apoptosis in human cancer cells” [file rsos171510supp1.docx]

Supporting Data for

**Synthesis of gypsogenin derivatives with capabilities to arrest cell cycle and induce apoptosis in human cancer cells**

Haochao Zhang^1,2^, Yanling Mu^2,3,4^, Fengling Wang^2,3,4^, Leling Song^1,2^, Jie Sun^2,3,4^, Yongjun Liu^2,3,4^, Jingyong Sun^2,3,4,^*

^1^ *School of Medicine and Life Sciences, University of Jinan-Shandong Academy of Medical Sciences, Jinan 250200, Shandong, China*

^2^ *Institute of Materia Medica, Shandong Academy of Medical Sciences, Jinan 250062, Shandong, China*

^3^ *Key Laboratory for Biotech-Drugs Ministry of Health, Jinan 250062, Shandong, China*

^4^ *Key Laboratory for Rare and Uncommon Diseases of Shandong Province, Jinan 250062, Shandong, China*

* *Corresponding author. Jinan 250062, Shandong, China. Tel. / Fax: +86-0531-8291-9963. E-mail address: sunjingyong08@hotmail.com*

**Supplementary Material**

**Contents**

[**Figure and Table** 2](#_Toc486601466)

[**IR(KBr) of compounds 2**-**9j** 6](#_Toc486601462)

[**Mass spectra of compounds 2**-**9j** 22](#_Toc486601464)

[**^1^H NMR(600 MHz) of compounds 2**-**9j** 30](#_Toc486601465)

[**^13^C NMR(150 MHz) of compounds 2**-**9j** 46](#_Toc486601466)

**Table 1**Anti-cancer activity of investgated compounds against human cancer cell lines.

| Compound | IC_50_ (μM) | | | | Compound | IC_50_ (μM) | | | |
| --- | --- | --- | --- | --- | --- | --- | --- | --- | --- |
|  | A549 | LOVO | SKOV3 | HepG2 |  | A549 | LOVO | SKOV3 | HepG2 |
| 1 | 19.60±4.50 | 15.90±1.87 | 20.67±3.77 | 22.18±2.62 | 8b | 28.83±6.78 | 26.83±2.44 | >30 | >30 |
| 2 | 30.86±3.26 | 14.36±2.21 | 21.20±2.13 | 24.71±3.15 | 8c | 27.77±5.14 | >30 | >30 | >30 |
| 3 | 17.70±2.49 | 12.35±1.34 | 18.51±1.18 | 19.15±2.21 | 8d | 25.70±2.68 | >30 | >30 | >30 |
| 4 | 3.10±1.14 | 2.97±1.13 | 10.04±1.38 | 9.71±2.06 | 8e | 15.77±2.37 | 12.36±2.35 | 25.63±1.14 | 27.88±2.42 |
| 5 | 28.23±2.04 | 12.42±1.03 | >30 | >30 | 8f | 15.03±3.48 | 11.14±1.87 | 24.59±2.72 | 26.91±2.74 |
| 6 | 26.50±1.77 | 5.31±1.26 | >30 | >30 | 8g | 14.16±8.70 | 10.88±1.32 | 27.38±3.49 | 28.17±2.88 |
| 7a | 17.08±2.32 | 13.49±2.86 | 16.23±2.35 | 19.14±2.53 | 9a | >30 | 25.98±1.65 | >30 | >30 |
| 7b | 15.57±2.17 | 10.44±2.73 | 15.74±1.44 | 17.50±2.48 | 9b | >30 | 24.36±1.48 | >30 | >30 |
| 7c | 7.32±1.28 | 7.59±1.63 | 10.10±2.19 | 12.33±2.63 | 9c | >30 | 24.06±1.17 | >30 | >30 |
| 7d | 7.04±1.54 | 6.18±1.46 | 9.88±1.54 | 11.68±1.33 | 9d | >30 | 24.65±2.05 | >30 | >30 |
| 7e | 11.05±1.87 | 5.24±1.69 | 17.34±1.24 | 16.58±1.84 | 9e | >30 | 28.54±2.37 | >30 | >30 |
| 7f | 10.55±2.10 | 4.96±1.64 | 15.56±1.68 | 13.14±1.61 | 9f | >30 | 25.36±2.43 | >30 | >30 |
| 7g | 9.24±1.53 | 3.59±2.04 | 13.16±2.96 | 12.55±1.41 | 9g | >30 | 24.55±1.42 | >30 | >30 |
| 7h | 8.36±3.97 | 22.37±3.08 | 24.77±2.78 | >30 | 9h | >30 | >30 | >30 | >30 |
| 7i | 8.04±3.65 | 28.93±2.89 | >30 | 29.61±2.44 | 9i | >30 | >30 | >30 | >30 |
| 7j | 9.47±2.81 | 26.42±3.25 | 28.74±3.24 | >30 | 9j | >30 | >30 | >30 | >30 |
| 8a | >30 | >30 | >30 | >30 | Cisplatin | 0.48±0.21 | 0.32±0.17 | 1.55±0.43 | 0.96±0.51 |

**Table 2** Apoptotic rate of cells treated with and without compounds 4 (5 µM) and 7g (5 µM) for 48 h.

|  | Live cells | Apoptotic cells | Necrotic cells |
| --- | --- | --- | --- |
| Control | 92.5 | 2.5 | 5.0 |
| Compound 4(5µM) | 54.1 | 18.9 | 27.0 |
| Compound 7g(5µM) | 68.6 | 17.1 | 14.3 |

**Scheme 1:** Reagents and conditions: (a) 2, 4-Dinitrophenylhydrazine, acetic acid, rt; (b) Hydroxylaminehydrochloride (NH_2_OH·HCl), pyridine, 105℃; (c) Acetic anhydride, pyridine, rt

**Scheme 2:** Reagents and conditions: (d) CH_2_Cl_2_, oxalyl chloride, secondary amine or primary alcohol, rt

**Scheme 3:** Reagents and conditions: (e) Ethyl alcohol absolute, 2.0 mol/L NaOH, 1.0 mol/L, rt

**Figure1**. The structure of gypsogenin

**Figure 2** (A) Morphological changes in LOVO cells treated with and without compound 4 for 48 h; (a) LOVO control cells; (b) LOVO cells treated with 5 μM for 48 h followed by morphological observation using Acridine Orange and Ethidium Bromide (AO/EB) cell staining method. (B) Effect of LOVO on cell cycle progression of colon cancer cells, (a) LOVO control cells; (b, c) LOVO cells treated with 5 and 10 μM for 48 h followed by analysis of cell cycle distribution using propidium iodide cell staining method. Cell population in each cell cycle phase was numerically depicted. Data represent one of three independent experiments.

**Figure 3** (A) Morphological changes in LOVO cells treated with compound 7g for 48 h; (a) LOVO control cells; (b) LOVO cells treated with 5 μM for 48 h followed by morphological observation using Acridine Orange and Ethidium Bromide (AO/EB) cell staining method. (B) Effect of LOVO on cell cycle progression of colon cancer cells; (a, b) LOVO cells treated with 5 and 10 μM for 48 h followed by analysis of cell cycle distribution using propidium iodide cell staining method. Cell population in each cell cycle phase was numerically depicted. Data represent one of three independent experiments.


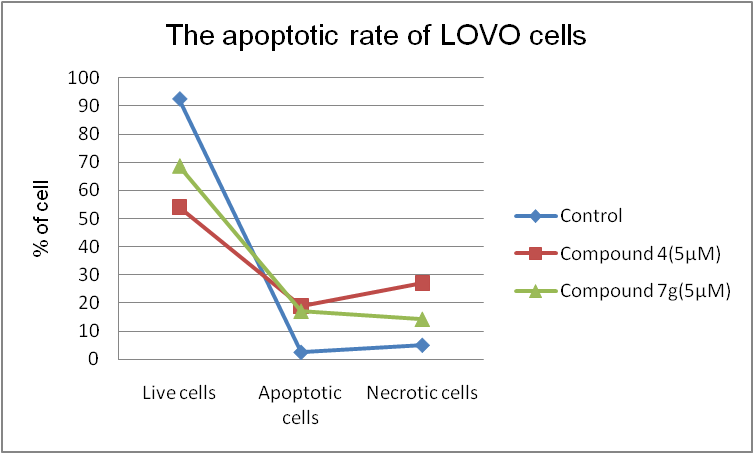


**Figure 4** Apoptotic rate of cells treated with and without compounds 4 (5 µM) and 7g (5 µM) for 48 h.

**IR (KBr) of compounds** **2**-**9j**

For compound **2**, IR spectra were recorded on a Thermo Nicolet Nexus 670 FT-IR spectrometer:

For compound **3**, IR spectra were recorded on a Thermo Nicolet Nexus 670 FT-IR spectrometer:

For compound **4**, IR spectra were recorded on a Thermo Nicolet Nexus 670 FT-IR spectrometer:

For compound **5**, IR spectra were recorded on a Thermo Nicolet Nexus 670 FT-IR spectrometer:

For compound **6**, IR spectra were recorded on a Thermo Nicolet Nexus 670 FT-IR spectrometer:

For compound **7a**, IR spectra were recorded on a Thermo Nicolet Nexus 670 FT-IR spectrometer:

For compound **7b**, IR spectra were recorded on a Thermo Nicolet Nexus 670 FT-IR spectrometer:

For compound **7c**, IR spectra were recorded on a Thermo Nicolet Nexus 670 FT-IR spectrometer:

For compound **7d**, IR spectra were recorded on a Thermo Nicolet Nexus 670 FT-IR spectrometer:

For compound **7e**, IR spectra were recorded on a Thermo Nicolet Nexus 670 FT-IR spectrometer:

For compound **7f**, IR spectra were recorded on a Thermo Nicolet Nexus 670 FT-IR spectrometer:

For compound **7g**, IR spectra were recorded on a Thermo Nicolet Nexus 670 FT-IR spectrometer:

For compound **7h**, IR spectra were recorded on a Thermo Nicolet Nexus 670 FT-IR spectrometer:

For compound **7i**, IR spectra were recorded on a Thermo Nicolet Nexus 670 FT-IR spectrometer:

For compound **7j**, IR spectra were recorded on a Thermo Nicolet Nexus 670 FT-IR spectrometer:

For compound **8a**, IR spectra were recorded on a Thermo Nicolet Nexus 670 FT-IR spectrometer:

For compound **8b**, IR spectra were recorded on a Thermo Nicolet Nexus 670 FT-IR spectrometer:

For compound **8c**, IR spectra were recorded on a Thermo Nicolet Nexus 670 FT-IR spectrometer:

For compound **8d**, IR spectra were recorded on a Thermo Nicolet Nexus 670 FT-IR spectrometer:

For compound **8e**, IR spectra were recorded on a Thermo Nicolet Nexus 670 FT-IR spectrometer:

For compound **8f**, IR spectra were recorded on a Thermo Nicolet Nexus 670 FT-IR spectrometer:

For compound **8g**, IR spectra were recorded on a Thermo Nicolet Nexus 670 FT-IR spectrometer:

For compound **9a**, IR spectra were recorded on a Thermo Nicolet Nexus 670 FT-IR spectrometer:

For compound **9b**, IR spectra were recorded on a Thermo Nicolet Nexus 670 FT-IR spectrometer:

For compound **9c**, IR spectra were recorded on a Thermo Nicolet Nexus 670 FT-IR spectrometer:

For compound **9d**, IR spectra were recorded on a Thermo Nicolet Nexus 670 FT-IR spectrometer:

For compound **9e**, IR spectra were recorded on a Thermo Nicolet Nexus 670 FT-IR spectrometer:

For compound **9f**, IR spectra were recorded on a Thermo Nicolet Nexus 670 FT-IR spectrometer:

For compound **9g**, IR spectra were recorded on a Thermo Nicolet Nexus 670 FT-IR spectrometer:

For compound **9h**, IR spectra were recorded on a Thermo Nicolet Nexus 670 FT-IR spectrometer:

For compound **9i**, IR spectra were recorded on a Thermo Nicolet Nexus 670 FT-IR spectrometer:

For compound **9j**, IR spectra were recorded on a Thermo Nicolet Nexus 670 FT-IR spectrometer:

**Mass spectra of compounds 2-9j**

ESI mass spectrum of compound **2** recorded on an AGILENT 1200 Capillary spectrometer:

ESI mass spectrum of compound **3** recorded on an AGILENT 1200 Capillary spectrometer:

ESI mass spectrum of compound **4** recorded on an AGILENT 1200 Capillary spectrometer:

ESI mass spectrum of compound **5** recorded on an AGILENT 1200 Capillary spectrometer:

ESI mass spectrum of compound **6** recorded on an AGILENT 1200 Capillary spectrometer:

ESI mass spectrum of compound **7a** recorded on an AGILENT 1200 Capillary spectrometer:

ESI mass spectrum of compound **7b** recorded on an AGILENT 1200 Capillary spectrometer:

ESI mass spectrum of compound **7c** recorded on an AGILENT 1200 Capillary spectrometer:

ESI mass spectrum of compound **7d** recorded on an AGILENT 1200 Capillary spectrometer:


ESI mass spectrum of compound **7e** recorded on an AGILENT 1200 Capillary spectrometer:

ESI mass spectrum of compound **7f** recorded on an AGILENT 1200 Capillary spectrometer:

ESI mass spectrum of compound **7g** recorded on an AGILENT 1200 Capillary spectrometer:

ESI mass spectrum of compound **7h** recorded on an AGILENT 1200 Capillary spectrometer:

ESI mass spectrum of compound **7i** recorded on an AGILENT 1200 Capillary spectrometer:

ESI mass spectrum of compound **7j** recorded on an AGILENT 1200 Capillary spectrometer:

ESI mass spectrum of compound **8a** recorded on an AGILENT 1200 Capillary spectrometer:

ESI mass spectrum of compound **8b** recorded on an AGILENT 1200 Capillary spectrometer:

ESI mass spectrum of compound **8c** recorded on an AGILENT 1200 Capillary spectrometer:

ESI mass spectrum of compound **8d** recorded on an AGILENT 1200 Capillary spectrometer:

ESI mass spectrum of compound **8e** recorded on an AGILENT 1200 Capillary spectrometer:

ESI mass spectrum of compound **8f** recorded on an AGILENT 1200 Capillary spectrometer:

ESI mass spectrum of compound **8g** recorded on an AGILENT 1200 Capillary spectrometer:

ESI mass spectrum of compound **9a** recorded on an AGILENT 1200 Capillary spectrometer:

ESI mass spectrum of compound **9b** recorded on an AGILENT 1200 Capillary spectrometer:

ESI mass spectrum of compound **9c** recorded on an AGILENT 1200 Capillary spectrometer:

ESI mass spectrum of compound **9d** recorded on an AGILENT 1200 Capillary spectrometer:

ESI mass spectrum of compound **9e** recorded on an AGILENT 1200 Capillary spectrometer:

ESI mass spectrum of compound **9f** recorded on an AGILENT 1200 Capillary spectrometer:

ESI mass spectrum of compound **9g** recorded on an AGILENT 1200 Capillary spectrometer:

ESI mass spectrum of compound **9h** recorded on an AGILENT 1200 Capillary spectrometer:

ESI mass spectrum of compound **9i** recorded on an AGILENT 1200 Capillary spectrometer:

ESI mass spectrum of compound **9j** recorded on an AGILENT 1200 Capillary spectrometer:

**^1^H NMR (600 MHz) of compounds 2-9j**

^1^H NMR spectrum of compound **2** recorded on a Bruker Avance III 600 spectrometer (600 MHz, pyridine-d_5_):

^1^H NMR spectrum of compound **3** recorded on a Bruker Avance III 600 spectrometer (600 MHz, pyridine-d_5_):

^1^H NMR spectrum of compound **4** recorded on a Bruker Avance III 600 spectrometer (600 MHz, pyridine-d_5_):

^1^H NMR spectrum of compound **5** recorded on a Bruker Avance III 600 spectrometer (600 MHz, pyridine-d_5_):

^1^H NMR spectrum of compound **6** recorded on a Bruker Avance III 600 spectrometer (600 MHz, chloroform-d):

^1^H NMR spectrum of compound **7a** recorded on a Bruker Avance III 600 spectrometer (600 MHz, pyridine-d_5_):

^1^H NMR spectrum of compound **7b** recorded on a Bruker Avance III 600 spectrometer (600 MHz, pyridine-d_5_):

^1^H NMR spectrum of compound **7c** recorded on a Bruker Avance III 600 spectrometer (600 MHz, pyridine-d_5_):

^1^H NMR spectrum of compound **7e** recorded on a Bruker Avance III 600 spectrometer (600 MHz, pyridine-d_5_):

^1^H NMR spectrum of compound **7f** recorded on a Bruker Avance III 600 spectrometer (600 MHz, pyridine-d_5_):

^1^H NMR spectrum of compound **7g** recorded on a Bruker Avance III 600 spectrometer (600 MHz, pyridine-d_5_):

^1^H NMR spectrum of compound **7h** recorded on a Bruker Avance III 600 spectrometer (600 MHz, pyridine-d_5_):

^1^H NMR spectrum of compound **7i** recorded on a Bruker Avance III 600 spectrometer (600 MHz, pyridine-d_5_):

^1^H NMR spectrum of compound **7j** recorded on a Bruker Avance III 600 spectrometer (600 MHz, pyridine-d_5_):

^1^H NMR spectrum of compound **8a** recorded on a Bruker Avance III 600 spectrometer (600 MHz, pyridine-d_5_):

^1^H NMR spectrum of compound **8b** recorded on a Bruker Avance III 600 spectrometer (600 MHz, pyridine-d_5_):

^1^H NMR spectrum of compound **8c** recorded on a Bruker Avance III 600 spectrometer (600 MHz, pyridine-d_5_):

^1^H NMR spectrum of compound **8d** recorded on a Bruker Avance III 600 spectrometer (600 MHz, pyridine-d_5_):

^1^H NMR spectrum of compound **8e** recorded on a Bruker Avance III 600 spectrometer (600 MHz, pyridine-d_5_):

^1^H NMR spectrum of compound **8f** recorded on a Bruker Avance III 600 spectrometer (600 MHz, pyridine-d_5_):

^1^H NMR spectrum of compound **8g** recorded on a Bruker Avance III 600 spectrometer (600 MHz, pyridine-d_5_):

^1^H NMR spectrum of compound **9a** recorded on a Bruker Avance III 600 spectrometer (600 MHz, chloroform-d):

^1^H NMR spectrum of compound **9b** recorded on a Bruker Avance III 600 spectrometer (600 MHz, chloroform-d):

^1^H NMR spectrum of compound **9c** recorded on a Bruker Avance III 600 spectrometer (600 MHz, chloroform-d):

^1^H NMR spectrum of compound **9d** recorded on a Bruker Avance III 600 spectrometer (600 MHz, chloroform-d):

^1^H NMR spectrum of compound **9e** recorded on a Bruker Avance III 600 spectrometer (600 MHz, chloroform-d):

^1^H NMR spectrum of compound **9f** recorded on a Bruker Avance III 600 spectrometer (600 MHz, chloroform-d):

^1^H NMR spectrum of compound **9g** recorded on a Bruker Avance III 600 spectrometer (600 MHz, chloroform-d):

^1^H NMR spectrum of compound **9h** recorded on a Bruker Avance III 600 spectrometer (600 MHz, chloroform-d):

^1^H NMR spectrum of compound **9i** recorded on a Bruker Avance III 600 spectrometer (600 MHz, chloroform-d):

^1^H NMR spectrum of compound **9j** recorded on a Bruker Avance III 600 spectrometer (600 MHz, chloroform-d):

**^13^C NMR (150 MHz) of compounds 2-9j**

^13^C NMR spectrum of compound **2** recorded on a Bruker Avance III 600 spectrometer (150 MHz, pyridine-d_5_):

^13^C NMR spectrum of compound **3** recorded on a Bruker Avance III 600 spectrometer (150 MHz, pyridine-d_5_):

^13^C NMR spectrum of compound **4** recorded on a Bruker Avance III 600 spectrometer (150 MHz, pyridine-d_5_):

^13^C NMR spectrum of compound **5** recorded on a Bruker Avance III 600 spectrometer (150 MHz, pyridine-d_5_):

^13^C NMR spectrum of compound **6** recorded on a Bruker Avance III 600 spectrometer (150 MHz, chloroform-d):

^13^C NMR spectrum of compound **7a** recorded on a Bruker Avance III 600 spectrometer (150 MHz, pyridine-d_5_):

^13^C NMR spectrum of compound **7b** recorded on a Bruker Avance III 600 spectrometer (150 MHz, pyridine-d_5_):

^13^C NMR spectrum of compound **7c** recorded on a Bruker Avance III 600 spectrometer (150 MHz, pyridine-d_5_):

^13^C NMR spectrum of compound **7d** recorded on a Bruker Avance III 600 spectrometer (150 MHz, pyridine-d_5_):

^13^C NMR spectrum of compound **7e** recorded on a Bruker Avance III 600 spectrometer (150 MHz, pyridine-d_5_):

^13^C NMR spectrum of compound **7f** recorded on a Bruker Avance III 600 spectrometer (150 MHz, pyridine-d_5_):

^13^C NMR spectrum of compound **7g** recorded on a Bruker Avance III 600 spectrometer (150 MHz, pyridine-d_5_):

^13^C NMR spectrum of compound **7h** recorded on a Bruker Avance III 600 spectrometer (150 MHz, pyridine-d_5_):

^13^C NMR spectrum of compound **7i** recorded on a Bruker Avance III 600 spectrometer (150 MHz, pyridine-d_5_):

^13^C NMR spectrum of compound **7j** recorded on a Bruker Avance III 600 spectrometer (150 MHz, pyridine-d_5_):

^13^C NMR spectrum of compound **8a** recorded on a Bruker Avance III 600 spectrometer (150 MHz, pyridine-d_5_):

^13^C NMR spectrum of compound **8b** recorded on a Bruker Avance III 600 spectrometer (600 MHz, pyridine-d_5_):

^13^C NMR spectrum of compound **8c** recorded on a Bruker Avance III 600 spectrometer (150 MHz, pyridine-d_5_):

^13^C NMR spectrum of compound **8d** recorded on a Bruker Avance III 600 spectrometer (150 MHz, pyridine-d_5_):

^13^C NMR spectrum of compound **8e** recorded on a Bruker Avance III 600 spectrometer (150 MHz, pyridine-d_5_):

^13^C NMR spectrum of compound **8f** recorded on a Bruker Avance III 600 spectrometer (150 MHz, pyridine-d_5_):

^13^C NMR spectrum of compound **8g** recorded on a Bruker Avance III 600 spectrometer (150 MHz, pyridine-d_5_):

^13^C NMR spectrum of compound **9a** recorded on a Bruker Avance III 600 spectrometer (150 MHz, chloroform-d):

^13^C NMR spectrum of compound **9b** recorded on a Bruker Avance III 600 spectrometer (150 MHz, chloroform-d):

^13^C NMR spectrum of compound **9c** recorded on a Bruker Avance III 600 spectrometer (150 MHz, chloroform-d):

^13^C NMR spectrum of compound **9d** recorded on a Bruker Avance III 600 spectrometer (150 MHz, chloroform-d):

^13^C NMR spectrum of compound **9e** recorded on a Bruker Avance III 600 spectrometer (150 MHz, chloroform-d):

^13^C NMR spectrum of compound **9f** recorded on a Bruker Avance III 600 spectrometer (600 MHz, chloroform-d):

^13^C NMR spectrum of compound **9g** recorded on a Bruker Avance III 600 spectrometer (150 MHz, chloroform-d):

^13^C NMR spectrum of compound **9h** recorded on a Bruker Avance III 600 spectrometer (150 MHz, chloroform-d):

^13^C NMR spectrum of compound **9i** recorded on a Bruker Avance III 600 spectrometer (150 MHz, chloroform-d):

^13^C NMR spectrum of compound **9j** recorded on a Bruker Avance III 600 spectrometer (150 MHz, chloroform-d):
